# Stromal CCL2 Signaling Promotes Mammary Tumor Fibrosis through Recruitment of Myeloid-Lineage Cells

**DOI:** 10.3390/cancers12082083

**Published:** 2020-07-28

**Authors:** Genevra Kuziel, Victoria Thompson, Joseph V. D’Amato, Lisa M. Arendt

**Affiliations:** 1 Program in Cancer Biology, University of Wisconsin-Madison, 1111 Highland Ave, Madison, WI 53705, USA; kuziel@wisc.edu; 2 Department of Comparative Biosciences, School of Veterinary Medicine, University of Wisconsin-Madison, 2015 Linden Drive, Madison, WI 53706, USA; victoria.thompson@wisc.edu (V.T.); joeydamato3@gmail.com (J.V.D.)

**Keywords:** CCL2, breast cancer, fibrocytes, obesity, macrophages, inflammation

## Abstract

Obesity is correlated with breast tumor desmoplasia, leading to diminished chemotherapy response and disease-free survival. Obesity causes chronic, macrophage-driven inflammation within breast tissue, initiated by chemokine ligand 2 (CCL2) signaling from adipose stromal cells. To understand how CCL2-induced inflammation alters breast tumor pathology, we transplanted oncogenically transformed human breast epithelial cells with breast stromal cells expressing CCL2 or empty vector into murine mammary glands and examined tumor formation and progression with time. As tumors developed, macrophages were rapidly recruited, followed by the emergence of cancer-associated fibroblasts (CAFs) and collagen deposition. Depletion of CD11b + myeloid lineage cells early in tumor formation reduced tumor growth, CAF numbers, and collagen deposition. CCL2 expression within developing tumors also enhanced recruitment of myeloid progenitor cells from the bone marrow into the tumor site. The myeloid progenitor cell population contained elevated numbers of fibrocytes, which exhibited platelet-derived growth factor receptor-alpha (PDGFRα)-dependent colony formation and growth in vitro. Together, these results suggest that chronic inflammation induced by CCL2 significantly enhances tumor growth and promotes the formation of a desmoplastic stroma through early recruitment of macrophages and fibrocytes into the tumor microenvironment. Fibrocytes may be a novel target in the tumor microenvironment to reduce tumor fibrosis and enhance treatment responses for obese breast cancer patients.

## 1. Introduction

Obesity is a global epidemic, and the World Health Organization estimates that obesity rates have nearly tripled since 1975 [1]. At the time of breast cancer diagnosis, obese women frequently have larger, higher grade tumors and an increased risk of metastasis, resulting in shorter disease-free and overall survival [2,3,4]. Breast tumors from obese women also demonstrate increased desmoplasia, compared to tumors from lean women [5]. Desmoplastic tumors, characterized by increased numbers of cancer associated fibroblasts (CAFs) and deposition of fibrillar collagens and extracellular matrix proteins within the stroma, are associated with diminished survival in breast cancer patients [6,7,8,9]. The origins of increased tumor collagen and desmoplasia, particularly in obesity, are not completely understood.

Obesity causes a state of chronic, macrophage-driven inflammation, as macrophages are recruited by necrotic adipocytes and form crown-like structures [10,11,12]. This inflammation is in large part driven by the expression of chemokine ligand 2 (CCL2), also referred to as monocyte chemoattractant protein-1, a chemokine which is first expressed by adipocytes in obese adipose tissue, and then by recruited macrophages [13,14]. In obese mice with germline deletion of CCL2 or its receptor, CCR2, macrophage recruitment to adipose tissue is significantly reduced [15,16]. Inflammation can also promote tumor growth, and increased CCL2 expression is correlated with more aggressive breast tumors as well as recruitment of tumor-associated macrophages [17,18,19]. Chronic inflammation has also been implicated in promoting adipose tissue fibrosis [20,21,22,23], which is characterized by deposition of collagen and other extracellular matrix proteins surrounding adipocytes. Within the mammary gland, elevated CCL2 expression driven by the mouse mammary tumor virus promoter in epithelial cells resulted in enhanced collagen deposition surrounding mammary ducts [24]. Within the breast tumor microenvironment, CCL2 expression by CAFs has been shown to enhance macrophage recruitment and promote cancer cell proliferation and cancer stem cells [25,26]. The link between inflammation and stroma formation, including the development of CAFs, in breast tumors is poorly understood. Although CAFs frequently express marker smooth muscle actin (SMA), CAFs are a heterogeneous population of cells that can be separated into subsets based on the expression of other markers, including fibroblast activation protein, fibroblast-specific protein-1, and platelet-derived growth factor receptor (PDGFRα and PDGFRβ) [27,28,29,30,31]. It is currently not clear how obesity promotes the growth of tumors with enhanced desmoplasia and potentially alters tumor CAF populations.

Understanding the impact of obesity on human breast tumors has been challenging to investigate due to the limited model systems to assess stromal changes during tumor progression. When transplanted into mouse mammary glands, human breast epithelial cells have limited ability to form epithelial structures [32,33]. However when transplanted with cells isolated from the stromal vascular fraction (SVF) of reduction mammoplasty tissues, breast stromal cells support the growth of normal human breast epithelial cells to produce ductal and alveolar structures containing polarized luminal and basal epithelial cells [34]. We have previously characterized a Human-In-Mouse (HIM) model to study how inflammation driven by CCL2 impacts breast tumor development and progression in vivo [18]. In the HIM model, SVF cells isolated from a patient with a body mass index in the normal range support the growth and progression of premalignant lesions from oncogenic human breast epithelial cells [18,35,36]. These SVF cells do not have an “activated” phenotype as has been observed in adipose stromal cells from obese women [37], allowing for investigation of the effects of CCL2 expression within the developing tumor microenvironment. Transplantation of human breast epithelial cells transduced with lentivirus containing oncogenes results in the stepwise progression of human breast epithelium through distinct stages of premalignancy [38]. These lesions as well as the resulting invasive carcinomas are histologically and molecularly similar to triple negative breast tumors, which lack expression of estrogen receptor, progesterone receptor, and human epidermal growth factor receptor 2 [35,36]. Using this mouse model, formation of tumors by human breast epithelial cells can be examined in vivo, in an inflammatory microenvironment with similarities to obese breast adipose tissue [18].

Here, we show that stromal CCL2 expression within developing mammary tumors enhanced end-stage tumor fibrosis. Myeloid-lineage CD11b + cells recruited early during tumor formation were critical for formation of CAFs and collagen deposition within tumors. With enhanced recruitment of macrophages, we also observed a significant increase in myeloid progenitor cells. Myeloid progenitor cells are a heterogenous cell population which give rise to multiple myeloid-lineage cell types, including bone marrow-derived fibrocytes, a cell population with characteristics of both macrophages and myofibroblasts. Fibrocytes have been identified in diseases characterized by both chronic inflammation and fibrosis [39,40,41]. Fibrocytes may be a novel target within the tumor microenvironment to limit tumor fibrosis. Understanding how CCL2 expression recruits myeloid-lineage cells to promote tumor progression and desmoplasia may lead to identification of new therapeutics to enhance treatment options for obese breast cancer patients.

## 2. Results

### 2.1. CCL2 Expression Enhances Collagen Deposition in End-Stage Tumors

**Figure 1:**
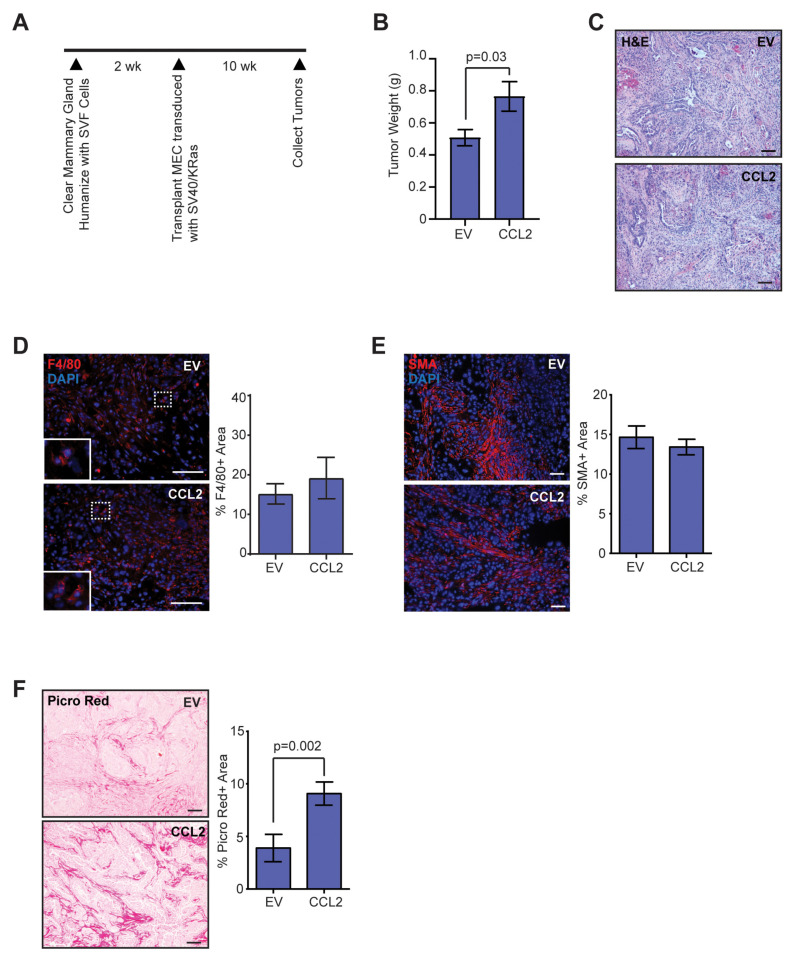
Elevated chemokine ligand 2 (CCL2) expression in stromal cells leads to increased collagen deposition in mammary tumors. (**A**) Schematic of Human-In-Mouse (HIM) transplants into recipient mouse mammary glands. (**B**) Tumor weight at end-stage from mice transplanted with SVF/EV or SVF/CCL2 stromal cells mixed with SV40ER/KRas^G12V^ transduced human breast epithelial cells (*n* = 12 tumors/group). (**C**) Representative H&E images of end-stage tumors. (**D**) Percent area of F4/80 + macrophages in end-stage tumors (*n* = 12 tumors/group). (**E**) Percent area of smooth muscle actin (SMA) + cells in end-stage tumors (*n* = 12 tumors/group). (**F**) Percent area of picrosirius red-stained collagen in end-stage tumors (*n* = 12 tumors/group). Statistical differences detected using Student’s *t*-test. Magnification bars = 50 µm.

Given the relationship between inflammation and adipose tissue fibrosis, we utilized the HIM model to examine how elevated macrophage-driven inflammation within developing mammary tumors affects collagen deposition. Inguinal mammary fat pads of 3-week-old nonobese diabetic/severe combined immunodeficiency (NOD/SCID) mice were cleared of endogenous mammary epithelium and humanized with SVF cells expressing empty vector (SVF/EV) or CCL2 (SVF/CCL2) (Figure 1A). Human breast epithelial cells were transduced with lentiviruses encoding KRas^G12V^ and SV40ER and implanted with SVF/EV or SVF/CCL2 stromal cells into humanized mammary fat pads 2 weeks later. Mammary tumors were collected after 10 weeks when tumors that formed in SVF/CCL2 humanized mammary glands reached 1 cm in diameter (end-stage). Similar to our previous studies [18], tumors grew significantly larger in SVF/CCL2 humanized glands compared to tumors from SVF/EV glands (*p* = 0.03; Figure 1B).

Although tumors grew faster in SVF/CCL2 humanized mammary glands, no significant differences were observed histologically among tumors in the SVF/EV and SVF/CCL2 groups (Figure 1C). To examine epithelial cells within SVF/EV and SVF/CCL2 tumors, tumor sections were stained with luminal epithelial cell marker cytokeratin 8 (CK8) and basal/myoepithelial cell marker cytokeratin 14 (CK14). Similar to human breast tumors, we observed distinct stromal and epithelial compartments (Figure 1C, Figure S1A). No significant differences were observed in the percent of cells that expressed CK8 or CK14 within SVF/EV or SVF/CCL2 tumors (Figure S1A). Isolated tumor cells demonstrated variable CCL2 expression, however no significant difference was observed among tumor cells from SVF/EV and SVF/CCL2 tumors (Figure S1B). SV40 expression was only observed within epithelial cells (Figure S1C). Epithelial cells expressing green fluorescent protein (GFP) from the KRas^G12V^ lentiviral construct were distinct from SMA + stromal cells within the tumors (Figure S1D). Using fluorescence in situ hybridization, we have previously shown that mouse-derived stromal cells are recruited to form CAFs within tumors, while human-derived stromal cells are limited [42]. Together, these data indicate that the epithelial component of the tumors is transformed human breast epithelial cells whereas the stromal compartment is mouse-derived stromal cells.

To understand the composition of the mouse stroma within these tumors, we examined recruitment of F4/80 + macrophages and SMA + CAFs. Although we previously observed significant increases in macrophages early in tumor development in glands humanized with SVF/CCL2 stromal cells [18], no differences were observed within the SVF/EV and SVF/CCL2 mammary tumors for F4/80 + macrophages at end-stage (*p* = 0.5, Figure 1D). Similarly, no differences were observed for SMA + CAFs within tumors at end-stage (*p* = 0.4, Figure 1E), suggesting that increased CCL2 expression within the microenvironment early in tumor development did not have lasting effects on either macrophage or CAF numbers in end-stage tumors. Given the association of inflammation with adipose tissue fibrosis, we examined collagen deposition within the end-stage tumors. We observed a significant increase in collagen measured using picrosirius red and Masson’s trichrome staining in the SVF/CCL2 tumors of mice compared to SVF/EV tumors (*p* = 0.002, Figure 1F, Figure S1E), suggesting that early inflammation promotes a more fibrous mammary tumor microenvironment, similar to what is observed clinically in breast tumors of obese women [5].

### 2.2. Temporal Changes in Macrophages and CAFs Are Observed in Developing SVF/CCL2 Mammary Tumors

**Figure 2:**
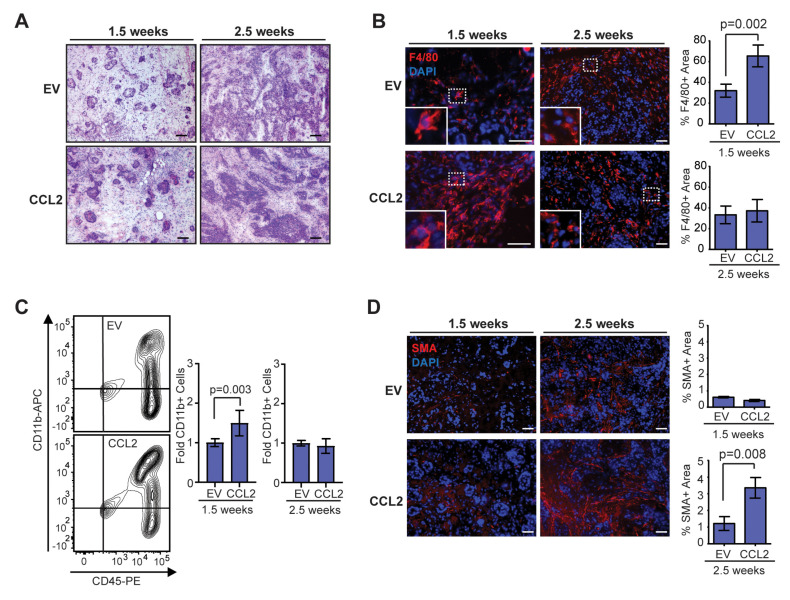
Stromal CCL2 overexpression in mouse mammary glands leads to time-dependent changes in the developing tumor microenvironment. (**A**) Representative H&E images of oncogenic epithelial cell growth at 1.5 and 2.5 weeks post-transplantation. (**B**) Percent area of F4/80 + macrophages in developing tumors at 1.5 and 2.5 weeks post-transplantation (*n* = 4 tumors/group; Student’s *t*-test). (**C**) CD45 + CD11b + monocytes/macrophages in the mammary gland at 1.5 and 2.5 weeks post-transplantation were quantified by flow cytometry (*n* = 5 EV, 7 CCL2, Mann–Whitney U test). (**D**) Percent area of SMA + cells in developing tumors at 1.5 and 2.5 weeks post-transplantation (*n* = 4 tumors/group; Student’s *t*-test). Magnification bars = 50 µm.

To investigate how early changes in tumor development lead to increased collagen deposition in end-stage SVF/CCL2 tumors, we examined early time points following transplantation of oncogenic breast epithelial cells. When transplanted in the HIM model, breast epithelial cells isolated from reduction mammoplasty tissue form alveoli with lumens surrounded by an inner layer of epithelial cells that express estrogen receptor alpha and an outer layer of basal/myoepithelial cells [34,38]. At 1.5 weeks following transplantation, we observed breast epithelial cells that filled the lumens surrounded by stromal cells in mammary glands humanized with either SVF/EV or SVF/CCL2 cells (Figure 2A). After 2.5 weeks, tumors were not yet palpable, however, disorganized epithelial cells could be observed surrounded by stromal cells (Figure 2A). Similar to our observations in end-stage tumors, we did not observe significant differences in the percentage of cells expressing CK8 or CK14 at either 1.5 or 2.5 weeks (Figure S2A,B). These results suggest that transplanted epithelial cells progressed through hyperplastic stages prior to invasive tumor growth.

Within the transplanted mammary glands at 1.5 weeks, we observed a significant increase in F4/80 + macrophages in the stroma of SVF/CCL2 transplants compared to SVF/EV transplants (*p* = 0.002, Figure 2B). The macrophages were primarily recruited to the transplant site, as no crown-like structures were observed within the surrounding mammary adipose tissue (data not shown). Interestingly, at 2.5 weeks post-transplant, F4/80 + cells were similar in both groups (Figure 2B). To further examine changes in macrophages, we quantified CD11b + cells from transplanted mammary glands using flow cytometry (Figure S2C–G). We observed a significant increase in CD11b + cells within SVF/CCL2 transplants at 1.5 weeks compared to SVF/EV transplanted mammary glands (*p* = 0.003, Figure 2C). This difference in CD11b + cells was no longer apparent at 2.5 weeks post-transplant (Figure 2C). In response to CCL2, myeloid-lineage cells are recruited from the bone marrow. To assess changes in CD11b + cells within the bone marrow, we quantified myeloid-lineage cells using flow cytometry (Figure S3A–E). No significant differences were observed in CD11b + cells within the bone marrow of SVF/EV and SVF/CCL2 mice at either 1.5 or 2.5 weeks post-transplant, although the percentage of CD11b + cells was decreased within the bone marrow from transplanted mice at 2.5 weeks compared to 1.5 weeks (Figure S3F,G), suggestive of increased CD11b + cell recruitment to the developing tumors. Together these data demonstrate that humanization of mouse mammary glands with CCL2 expressing stromal cells enhances macrophage recruitment early in tumor formation.

While CAFs have been examined extensively in invasive ductal carcinoma, CAFs also play a role in progression of ductal carcinoma in situ to invasive ductal carcinoma [43,44]. Within developing tumors of our HIM mice, limited SMA expression was observed in the stroma of both SVF/EV and SVF/CCL2 transplants at 1.5 weeks, and no significant differences were observed between the two groups (Figure 2D). However, at 2.5 weeks following transplantation, we observed a significant increase in SMA + CAFs within the SVF/CCL2 transplant sites compared to SVF/EV transplants (*p* = 0.008, Figure 2D). Together, these data suggest that early increases in macrophage-driven inflammation lead to enhanced CAF formation within developing tumors.

### 2.3. Depletion of CD11b + Cells Early in Tumor Formation Reduces Tumor Growth and Collagen Deposition

**Figure 3:**
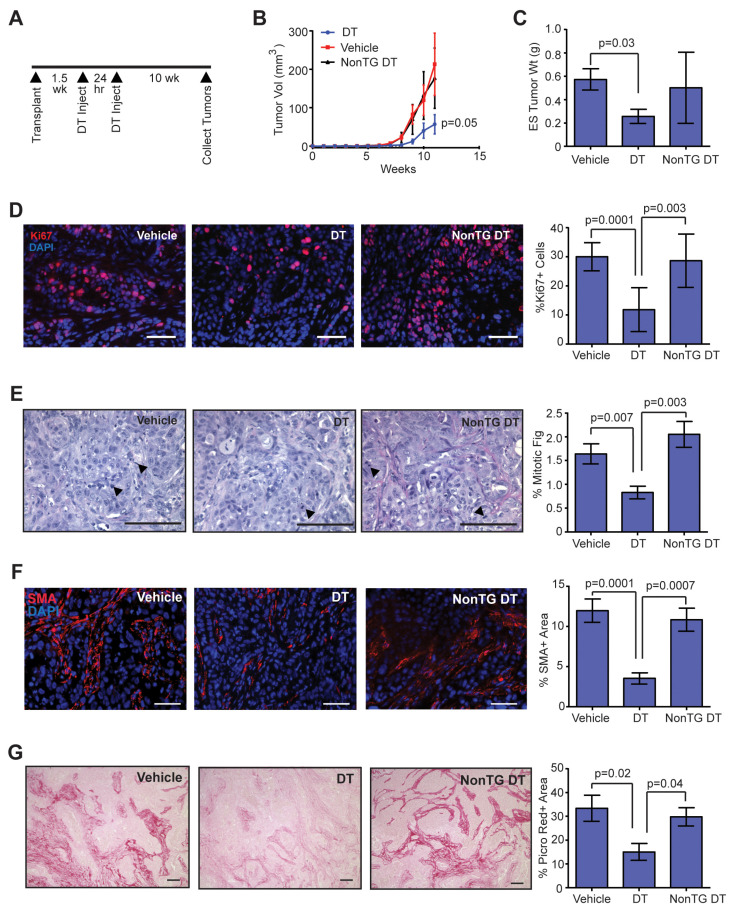
Depletion of CD11b + cells early in tumor development reduces tumor growth, collagen-producing cells, and collagen deposition within tumors. (**A**) DTR-CD11b (Mac/SCID) transgenic mice and non-transgenic littermates (NonTG) were humanized with stromal vascular fraction (SVF)/CCL2 cells, then transplanted with oncogenic human breast epithelial cells. Mice were injected with two doses of vehicle or diphtheria toxin (DT) at 1.5 weeks post-transplantation to reduce CD11b + cells. (**B**) Tumor growth curves following epithelial cell transplantation and treatment with vehicle or DT (*n* = 8 tumors/group; two-way ANOVA analysis with Tukey’s multiple comparisons test). (**C**) Tumor weight measured at end-stage (*n* = 8 tumors/group). (**D**) Percent of cells expressing proliferation marker Ki67 in end-stage tumors (*n* = 8 tumors/group). (**E**) Percent of mitotic figures in end-stage tumors (*n* = 8 tumors/group). (**F**) Percent area of SMA + cells in end-stage tumors (*n* = 8 tumors/group). (**G**) Percent area of picrosirius red stained collagen in end-stage tumors (*n* = 8 tumors/group). Statistical differences detected using one-way ANOVA analysis with Tukey’s multiple comparisons test. Magnification bars = 50 µm.

To examine the relationship between the influx of macrophages and the other stromal changes observed during tumor progression, we depleted CD11b + cells using DTR-CD11b (Mac/SCID) mice. In these mice, expression of the simian diphtheria toxin receptor is regulated by the CD11b promoter, and upon treatment with diphtheria toxin (DT), monocytes and macrophages are selectively depleted [45,46]. We transplanted human breast epithelial cells transduced with SV40ER and KRas^G12V^ into inguinal mammary glands of Mac/SCID mice humanized with SVF/CCL2 stromal cells. At 1.5 weeks following transplantation, mice were treated with two doses of DT administered 24 h apart to reduce the number of CD11b + cells present at this early time point (Figure 3A). Transgenic mice treated with DT demonstrated significantly reduced CD11b + cells within the bone marrow and within transplant sites 24 h after the second injection of DT, compared to either non-transgenic mice treated with DT or vehicle-treated transgenic mice (Figure S4A–D). We have previously shown that this reduction of CD11b + cells lasts for at least 7 days within the mammary gland [18].

Treatment of Mac/SCID mice with DT resulted in significantly reduced tumor growth compared to tumors from vehicle-treated Mac/SCID mice or non-transgenic littermates treated with DT (*p* = 0.05; Figure 3B). At end-stage, tumors from DT-treated Mac/SCID mice had significantly reduced tumor weights compared to vehicle-treated Mac/SCID mice (*p* = 0.03; Figure 3C). Consistent with slower tumor growth, we observed significantly reduced Ki67 staining (Figure 3D) and mitotic figures (Figure 3E) in end-stage tumors of DT-treated Mac/SCID mice compared to either vehicle-treated Mac/SCID mice or DT-treated non-transgenic mice. When Ki67 staining was examined further, the majority of Ki67 positive cells were observed in the GFP + human epithelial cell population, not in the stromal compartment (Figure S4E). Although we observed significantly decreased tumor cell proliferation within the end-stage tumors of DT-treated Mac/SCID mice, we did not observe significant differences in the ratio of CK8 or CK14 expressing tumor cells (Figure S4F), suggesting that DT treatment did not significantly alter the composition of tumor cell populations. Together, these results suggest that depletion of CD11b + cells during early tumor development resulted in lasting changes in tumor cell proliferation and growth at end-stage.

Interestingly, we observed increased areas of tumor cells within the end-stage tumors of DT-treated Mac/SCID mice compared to tumors of either vehicle-treated Mac/SCID or non-transgenic DT-treated mice (Figure S4F), suggestive of changes within the stroma of these tumors. To quantify changes in macrophages that might be present within end-stage tumors of DT-treated Mac/SCID mice, we stained tumor sections for F4/80. No significant differences were observed in F4/80 + macrophages within end-stage tumors of DT-treated Mac/SCID mice compared to vehicle-treated Mac/SCID or non-transgenic DT-treated mice (Figure S4G), indicating that the macrophage population had recovered in end-stage tumors following depletion with DT. However, SMA + CAFs were significantly reduced in tumors of DT-treated Mac/SCID mice compared to either vehicle-treated Mac/SCID or non-transgenic DT-treated mice (Figure 3F), indicating an overall loss in recruitment and/or differentiation of CAFs within these tumors. Consistent with reduced numbers of CAFs, DT-treated Mac/SCID mice demonstrated significantly reduced collagen deposition within end-stage tumors compared to either vehicle-treated Mac/SCID or non-transgenic DT-treated mice, quantified by staining for picrosirius red (Figure 3G), which stains both fibrillar and non-fibrillar collagens [47], and collagen I (Figure S4H). Together, these results demonstrate that depletion of CD11b + cells early in mammary tumor development resulted in reduced CAFs and collagen deposition.

### 2.4. Myeloid Progenitor Cell Population Is Increased in Early Tumor Development and Is Enriched for Fibrocytes

**Figure 4:**
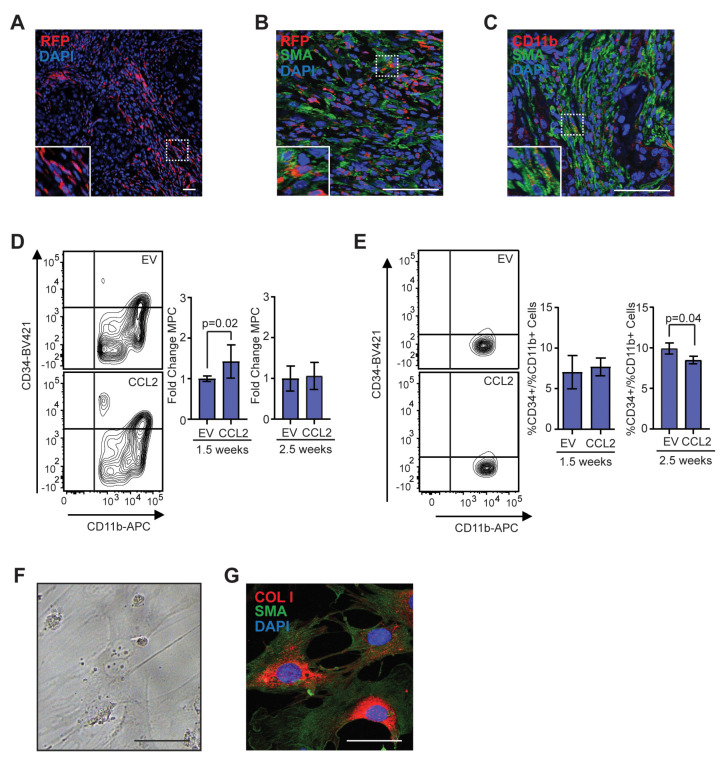
Population of CD11b + myeloid progenitor cells differentiate into SMA + stromal cells within tumors and in vitro. (**A**) Representative image of red fluorescent protein (RFP) + stromal cells in tumor from CCR2-RFP heterozygous SCID mouse. (**B**) RFP + SMA + double positive cells within tumor stroma. (**C**) CD11b + SMA + double positive cells within tumor stroma. (**D**) CD45 + CD11b + CD34+ myeloid progenitor cells in the mammary gland at 1.5 (*n* = 5 empty vector (EV), 7 CCL2) and 2.5 weeks (*n* = 5 EV, 7 CCL2) post-transplantation were quantified by flow cytometry. (**E**) CD45 + CD11b + CD34 + myeloid progenitor cells in the bone marrow at 1.5 weeks (*n* = 6 EV, 7 CCL2) and 2.5 weeks (*n* = 3 mice/group) post-transplantation were quantified by flow cytometry. (**F**) Representative brightfield image of colony formed by CD45 + CD11b + CD34 + myeloid progenitor cells isolated using fluorescence-activated cell sorting (FACS). (**G**) Colonies in culture co-stained with SMA and collagen I. Statistical differences determined by Mann–Whitney U test. Magnification bars = 50 µm.

CCR2 is expressed on multiple cell types of the myeloid lineage, including monocytes/macrophages, dendritic cells, and natural killer cells, and plays a critical role in recruitment of these cells from the bone marrow [48]. To investigate the recruitment of CCR2 + myeloid-lineage cells into tumors, we used SVF/CCL2 stromal cells to humanize the inguinal mammary glands of heterozygous CCR2/SCID mice, which have the CCR2 coding sequence replaced with red fluorescent protein (RFP). We then transplanted human breast epithelial cells transduced with SV40ER and KRas^G12V^ into the humanized mammary glands. Within end-stage tumors, we observed that CD11b + cells comprised approximately 31.5% of the tumor cells, while F4/80 + cells were approximately 21.8% (Figure S5A,B). The majority of CD11b + cells expressed RFP (78.7%), while 20.2% expressed CD11b alone (Figure S5C). These RFP-cells may be consistent with neutrophils [48]. We also observed RFP + cells within the tumor stroma, with a population of RFP + cells having an elongated morphology similar to CAFs (inset, Figure 4A). To investigate these cells further, we co-labeled tumors for RFP and SMA. Within the tumor stroma, we observed elongated cells that co-expressed both markers with a frequency of 8.7 ± 3.9% of SMA + cells (Figure 4B, Figure S5D,E). Similarly, we observed elongated cells within the tumor stroma that co-expressed CD11b and SMA (Figure 4C, Figure S5F).

Within the myeloid lineage, fibrocytes are recruited from the bone marrow under conditions of chronic inflammation and fibrosis and differentiate into myofibroblasts [39,40,41]. Fibrocytes are identified by expression of myeloid progenitor cell markers coupled with markers for myofibroblasts, including SMA and collagen I. To examine changes in the myeloid progenitor cell population during tumor development, we humanized NOD/SCID mice with either SVF/EV or SVF/CCL2 cells, then transplanted human breast epithelial cells transduced with SV40ER and KRas^G12V^ into the humanized mammary glands. At 1.5 weeks following transplantation, we observed a significant increase in CD45 + CD11b + CD34 + myeloid progenitor cells within SVF/CCL2 transplants compared to SVF/EV transplants (*p* = 0.02; Figure 4D). Similar to our observations of total CD11b + cells (Figure 2C), no significant difference in myeloid progenitor cells was observed between SVF/EV and SVF/CCL2 at 2.5 weeks following transplantation (Figure 4D). To assess changes in myeloid progenitor cells systemically, we isolated bone marrow cells from mice with transplanted mammary glands. At 1.5 weeks following transplantation, no significant difference was observed in myeloid progenitor cells within the bone marrow of SVF/EV and SVF/CCL2 transplanted mice (Figure 4E). However, at 2.5 weeks following transplantation, SVF/CCL2 transplanted mice had significantly reduced myeloid progenitor cells within the bone marrow (*p* = 0.04; Figure 4E), suggestive of recruitment of myeloid progenitor cells out of the bone marrow niche. Taken together, these data suggest that myeloid progenitor cells are recruited from the bone marrow to the transplants of SVF/CCL2 humanized glands early in tumor formation.

To further investigate the presence of fibrocytes, CD45 + CD11b + CD34 + myeloid progenitor cells and CD45 + CD11b + CD34-monocytes/macrophages were isolated from transplanted HIM mammary glands using fluorescence-activated cell sorting (FACS). When plated in culture, a portion of the myeloid progenitor cell population formed adherent colonies with cellular morphology similar to myofibroblasts, while no colony formation was observed within the monocyte/macrophage population (Figure 4F). This data suggests that a subset of cells within the myeloid progenitor cell population can differentiate into adherent cells and proliferate. These adherent colonies grown in culture co-expressed SMA and collagen I (Figure 4G), consistent with fibrocyte colony formation. The colonies that formed were negative for expression of macrophage marker, F4/80 (data not shown). These data suggest that fibrocytes may contribute to the CAF population within the developing mammary tumors through differentiation into SMA + and collagen I + stromal cells.

### 2.5. Fibrocytes Are PDGFR Responsive Cells

**Figure 5:**
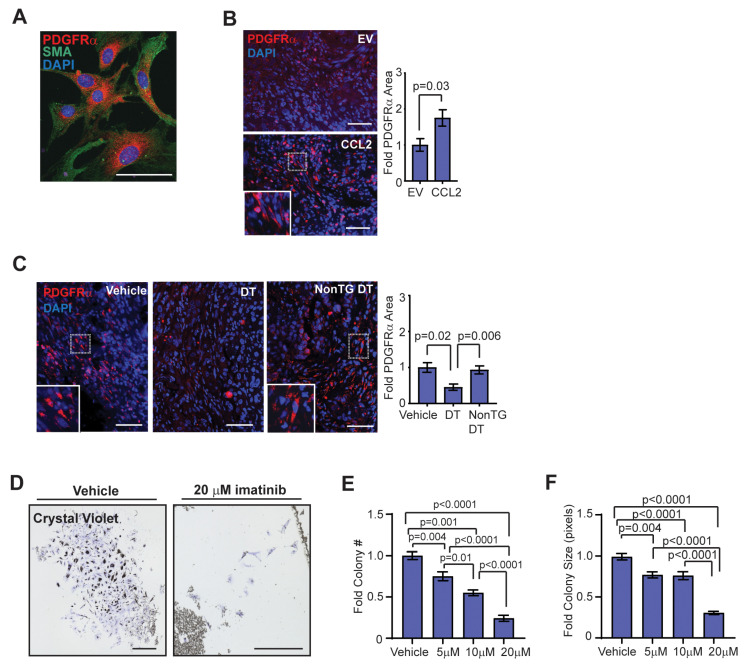
Fibrocytes express platelet-derived growth factor receptor (PDGFR)α and have a dose-dependent response to imatinib treatment. (**A**) Colonies in culture co-stained with PDGFRα and SMA. (**B**) Fold change in percent area of PDGFRα expression in end-stage tumors of SVF/EV and SVF/CCL2 mice (*n* = 8 tumors/group; Student’s *t*-test). (**C**) Fold change in percent area of PDGFRα expression in end-stage tumors of Mac/SCID mice (*n* = 12 tumors/group; one-way ANOVA analysis with Tukey’s multiple comparison test). (**D**) Crystal violet-stained CD11b + colonies grown from magnetic bead-sorted mammary gland cells treated with vehicle or 20 µM imatinib. (**E**) Fibrocyte colony number with vehicle, 5 µM, 10 µM, and 20 µM imatinib treatment (*n* = 3 mice/treatment group; one-way ANOVA analysis with Tukey’s multiple comparisons test). (**F**) Fibrocyte colony size with vehicle, 5 µM, 10 µM, and 20 µM imatinib treatment (*n* = 3 mice/treatment group; Kruskal–Wallis analysis with Dunn’s multiple comparisons test). Magnification bars = 50 µm.

Previously, fibrocytes have been shown to express PDGFRα and migrate in response to PDGF-AA signaling [49]. Consistent with this, fibrocyte colonies grown in culture from FACS-isolated mammary myeloid progenitor cells co-expressed PDGFRα and SMA (Figure 5A). To examine PDGFRα expression within the tumor stroma, sections from end-stage tumor sections from the HIM model were stained for PDGFRα. Tumors from SVF/CCL2 humanized glands demonstrated significantly increased PDGFRα expression compared to tumors from SVF/EV humanized glands (*p* = 0.03; Figure 5B). Depletion of CD11b + cells in SVF/CCL2 HIM transplanted mice resulted in a significant reduction of PDGFRα expression within the stroma of end-stage tumors compared to vehicle-treated Mac/SCID or non-transgenic DT-treated mice (Figure 5C). Together, these results indicate that early increases in CD11b + cells during tumor formation lead to increased PDGFRα + stromal cells in the tumor microenvironment.

To assess the role of PDGFRα in regulating fibrocyte differentiation and growth, we utilized imatinib mesylate, a clinically utilized tyrosine kinase inhibitor of PDGFR, stem cell factor (c-Kit), and Abelson non-receptor tyrosine kinase (c-Abl) that has shown some promise in the treatment of fibrotic diseases [50,51,52]. CD11b + cells were sorted from mammary glands using magnetic beads, and sorted cells were then treated in culture with increasing doses of imatinib. After 10 days of treatment with vehicle, 5 µM, 10 µM, or 20 µM imatinib, fibrocyte colonies were fixed and stained with crystal violet (Figure 5D). Both colony number and colony size were decreased following treatment with imatinib in a dose-dependent manner (Figure 5E,F). These results suggest that treatment with imatinib inhibited fibrocyte colony formation through either reduced cellular proliferation or increased apoptosis. In contrast, when isolated CD11b + cells were treated in culture with the selective c-kit inhibitor ISCK03, inhibition of colony number and size were only observed at the 20 µM dose (Figure S5G). When isolated CD11b + cells were plated in serum-free media supplemented with vehicle or up to 100 ng/mL PDGF-AA, no colony formation was observed (data not shown), suggesting that PDGFRα signaling is not sufficient for fibrocyte colony formation and growth. Together, these results suggest that targeted inhibition of fibrocytes may reduce CCL2-induced desmoplasia within breast tumors to enhance chemotherapeutic efficacy.

## 3. Discussion

Obese mammary adipose tissue demonstrates both increased macrophage-driven inflammation and adipose tissue fibrosis [5,12,18]. Obesity is also correlated with desmoplasia in human breast tumors [5]. Understanding the relationship between obesity-associated inflammation and tumor desmoplasia is challenging, because obesity induces both local and systemic changes that could contribute to fibrosis. Here, we use the HIM model to examine inflammation induced by CCL2, which is significantly increased in obese adipose tissue [13,14], and the consequences within developing mammary tumors. A strength of this model is that transformed human breast epithelial cells progress through stages of breast cancer growth in a similar time frame, allowing for temporal examination of early tumor progression. We observed that elevated recruitment of macrophages into developing tumors led to increased collagen deposition within end-stage tumors (Figure S5H). This was associated with an increase in myeloid progenitor cells, containing fibrocytes, a cell population which expresses markers for both myeloid-lineage immune cells and myofibroblasts. Although fibrocytes are a relatively rare immune cell population [53], these cells may be enhanced in the context of obesity, as increases in circulating myeloid progenitor cells have been observed in obese patients [54]. In cancer, fibrocytes have been found to contribute to tumor fibrosis as well as resistance to anti-angiogenic therapies [55,56]. Fibrocytes may be an important therapeutic target to reduce tumor desmoplasia and treatment-resistance in obese patients with breast cancer.

Within the tumor microenvironment, CAFs comprise a large portion of breast tumor stroma. CAFs are a heterogeneous population of cells, and CAF populations expressing different markers may arise from various cells of origin [29,57,58]. Although not well-examined in the context of cancer, fibrocytes may contribute to CAF populations. Within tumors of the HIM model mice, we observed SMA + stromal cells that also expressed CD11b and CCR2, suggestive of differentiating fibrocytes. Fibrocytes within tissues are challenging to identify, as specific markers do not exist for this cell population. Fibrocyte cells express markers consistent with myeloid progenitor cells, which are thought to be the cells of origin [59]. However, following differentiation, fibrocytes may lose immune cell markers [60,61] and become indistinguishable from resident tissue fibroblasts [62]. Consistent with these observations, RFP expression controlled by the CCR2 promoter may be lost during differentiation of fibrocytes within tumors. In breast cancer specimens, CD34 expression is reduced and SMA expression is increased within the stroma of high grade ductal carcinoma in situ [63,64], which may reflect differentiation of fibrocytes within the developing tumor stroma. Although we observed that fibrocytes expressed PDGFRα in culture, PDGFRα is also expressed by mammary fibroblasts [65], and this marker may identify CAFs arising from multiple origins. Lineage tracing approaches to examine fibrocytes in wound healing [62], such as the Vav-Cre model, which directs cre recombinase expression under control of the promoter for pan-hematopoietic cell-specific protein Vav-1, may enhance the identification of fibrocyte-derived CAF populations.

Cancer has been described as a wound that will not heal [66]. In the context of wound healing, macrophages secrete multiple soluble mediators that stimulate local and recruited myofibroblasts to differentiate and facilitate wound closure and synthesis of extracellular matrix [67,68]. This function of macrophages may be critical in developing tumors to form the supportive tumor stroma. We observed that early depletion of CD11b + myeloid lineage cells resulted in the disruption of CAF formation and collagen deposition within the end-stage tumor stroma, suggesting a critical role for these myeloid lineage cells in early tumor growth that is not replicated by tumor cells. Similar results were observed with the growth of Lewis lung carcinoma cells in macrophage-deficient *op/op* mice [69]. Macrophages can also produce collagen family members [70] and have themselves been implicated in collagen remodeling in tumors [70,71], as well as during mammary gland development and involution [72,73]. We also observed the emergence of CD11b + fibrocytes, which may also enhance collagen deposition within developing tumors. Macrophages may promote fibrocyte differentiation through local secretion and activation of TGFβ1 [74,75]. Similar to macrophages, CAFs within the tumor microenvironment may enhance fibrocyte differentiation, potentially though secretion of the proteoglycan, lumican [76]. Although myeloid progenitor cells are thought to be the cells of origin of fibrocytes, others have described a macrophage-to-myofibroblast transition in renal fibrosis [77,78,79], indicating that multiple pathways may exist for myeloid lineage cell contribution to the CAF population. Increased understanding of interactions among stromal cells may improve strategies to therapeutically target the tumor microenvironment.

Within the tumor microenvironment, tumor-associated macrophages are thought to promote tumor growth through angiogenesis as well as through secretion of multiple cytokines implicated in enhancing aggressive tumor cell populations [80,81,82]. In breast cancer, CD68 + tumor-associated macrophages are correlated with Ki67 expression in epithelial cells [83], suggesting that increased numbers of macrophages enhance tumor cell proliferation. Following early depletion of CD11b + myeloid lineage cells, we observed a significant reduction in tumor cell expression of Ki67, decreased numbers of mitotic figures and diminished tumor growth rates. It is tempting to speculate that depletion of macrophages during tumor development had a direct impact on tumor cell populations, possibly through secretion of multiple inflammatory cytokines. However, this depletion of myeloid lineage cells also led to reductions in CAF number and collagen deposition within end-stage tumors, while macrophage number was not significantly different at end-stage. CAFs have also been implicated in promoting cancer cell proliferation within tumors [84,85]. Although both tumor-associated macrophages and CAFs secrete factors that enhance tumor cell proliferation, the loss of collagen could also significantly impact tumor growth. Tumor stiffening due to increased extracellular matrix deposition and collagen crosslinking has been implicated in promoting proliferation and invasion of mammary tumor cells [86,87] through mechanical signaling [88]. Further studies are necessary to determine whether the reduced proliferation of tumor cells was a direct or indirect effect of myeloid lineage depletion. However, these studies suggest that chemoprevention strategies targeting the microenvironment early in tumor formation could reduce the aggressiveness of tumors that form regardless of genetic abnormalities present within the tumor cells.

Imatinib mesylate (Gleevec) is a clinically available receptor tyrosine kinase inhibitor that is used to treat chronic myeloid leukemia patients long-term with limited side effects. Unfortunately, phase I clinical trials suggested that imatinib alone or in combination with chemotherapeutic agents did not significantly improve survival outcomes in patients with treatment-resistant metastatic breast cancer [89,90,91,92,93,94,95]. However, imatinib has significant anti-fibrotic effects through inhibition of PDGFR signaling as well as by blocking c-Abl upregulation of TGFβ1 pathways [96], resulting in effects such as reduced human breast fibroblast-mediated collagen deposition [97]. In a preclinical model of pulmonary fibrosis, imatinib reduced recruitment of fibrocytes to the lungs [49], and we observed that imatinib also decreased the ability of fibrocytes to form colonies in vitro, suggesting that imatinib may inhibit fibrocyte-mediated fibrosis. Currently, imatinib is in clinical trials for treatment of multiple fibrotic conditions [96,98,99]. Obesity enhances adipose tissue fibrosis, and in a preclinical model, treatment of obese mice with imatinib significantly reduced inflammation, enhanced adipogenesis, and diminished adipose tissue fibrosis [100,101]. Obese breast cancer patients may benefit from adjuvant treatment with imatinib to reduce fibrosis within breast tumors. Further studies to identify the contribution of fibrocytes to CAF populations with breast tumors may lead to improved treatment strategies to reduce desmoplasia in breast tumors of obese patients and enhance chemotherapeutic responses.

## 4. Materials and Methods

### 4.1. Human Breast Tissue

All human breast tissues were obtained in compliance with the laws and institutional guidelines, as approved by the Institutional Review Board at the University of Wisconsin-Madison (Human Subjects Assurance Number: FWA00005399). De-identified, non-cancerous breast tissues were obtained from patients undergoing elective reduction mammoplasty with informed consent through the BioBank (Carbone Cancer Center, University of Wisconsin-Madison, Madison, WI, USA). These studies were categorized as “not human subjects research.” Patient data were limited to date of service, body mass index, and age.

### 4.2. Transgenic Mice

All animal procedures were conducted in compliance with a protocol approved by the University of Wisconsin Institutional Animal Care and Use Committee and housed and handled in accordance with the Guide for Care and Use of Laboratory Animals in AAALAC accredited facilities (Animal Welfare Assurance Number: D16-00239). NOD/SCID (001303), SCID (001913), DTR CD11b-EGFP (008547), and CCR2-RFP (017586) mice were purchased from Jackson Laboratory (Bar Harbor, ME, USA). DTR CD11b and CCR2-RFP mice were crossed with SCID mice to generate DTR CD11b-EGFP/SCID (Mac/SCID) and CCR2-RFP/SCID (CCR2/SCID) mice homozygous for the SCID transgene. Genotyping was performed by Transnetyx (Cordova, TN, USA). Mice were given food and water ad libitum.

### 4.3. Human-in-Mouse Model

SVF/EV and SVF/CCL2 breast stromal cell lines were generated from the SVF of human reduction mammoplasty tissue as described [18]. To humanize mice, endogenous mammary epithelium was removed from the fourth mammary glands of 3-week-old female NOD/SCID, Mac/SCID, or CCR2/SCID mice and either SVF/EV or SVF/CCL2 cells were injected into the fat pad as described [102]. Two weeks following humanization, epithelial cells were isolated from reduction mammoplasty tissues, transduced overnight with lentiviruses encoding oncogenes KRas^G12V^ and SV40ER, and transplanted into the humanized area of mammary fat pads with SVF/EV or SVF/CCL2 cells, as previously described [18,36]. Briefly, lentiviral particles encoding pLenti 7.3 vector (ThermoFisher; Waltham, MA, USA; V53406), pLenti-KRas^G12V^-GFP, and pLenti-SV40 ER-blasticidin were generated and concentrated as described [36]. Reduction mammoplasty tissues were enzymatically digested as described [38,102], the epithelial organoids were dissociated to single cells and transduced in suspension with lentivirus with a multiplicity of infection of 3 [18,36]. Transduced epithelial cells (1 × 10^5^/gland) were mixed with either SVF/EV or SVF/CCL2 cells (2.5 × 10^5^/gland) in a 1:1 mixture of 2 mg/mL rat tail collagen (Corning Inc., Corning, NY, USA; 354236) and Matrigel (Corning, Inc; Corning, NY, USA; 35428) and injected into the humanized fat pads. Transplants were collected at early time points of 1.5 or 2.5 weeks following transplantation or at end-stage when tumors reached 1 cm in diameter. For CD11b + cell depletion, mice were treated with 12.5 mg/kg of diphtheria toxin (DT, Sigma-Aldrich St. Louis, MO, USA; D0564) in 2 doses injected subcutaneously 24 h apart starting 1.5 weeks after epithelial cell transplant. To examine depletion of CD11b + cells, Mac/SCID mice were treated with vehicle or DT, and mammary tissue and bone marrow were collected 24 h after the second dose. HIM model experiments were repeated 3 times with epithelial cells isolated from 3 different reduction mammoplasty samples.

### 4.4. Isolation of Cells From Mammary Glands

Mammary tissue collected 1.5 and 2.5 weeks post-transplant and end-stage tumors were mechanically minced then enzymatically dissociated for 1 h at 37 °C in media composed of DMEM/F-12 (Corning Inc.; Corning, NY, USA; 10-090-CV) supplemented with 10 μg/mL insulin (Sigma-Aldrich; I0516), 0.5 μg/mL hydrocortisone (Sigma-Aldrich; St. Louis, MO, USA; H0888), 10 ng/mL human epidermal growth factor (hEGF, Sigma-Aldrich; E9644), 5% calf serum, and 1% antibiotic/antimycotic solution (Corning, Inc.; Corning, NY, USA; 30-004-CI) with 1.5 mg/mL collagenase A (Millipore Sigma, St. Louis, MO, USA; COLLA-RO) and 125 U/mL hyaluronidase (Sigma-Aldrich; St. Louis, MO, USA; H3506). Following digestion, the tissue was left to settle at room temperature for 10 min. The surface lipid layer was discarded. Red blood cells were lysed using ACK Lysing Buffer (Lonza, Basel, Switzerland; 10-548E). Epithelial organoids were dissociated to single cells using 0.25% Trypsin, 0.1% EDTA (Corning; 25-053-CI) at 37 °C for 10 min followed by 50 µg/mL DNase (Millipore Sigma; St. Louis, MO, USA; 10104159001). Cells were then passed through a 40 µm Falcon cell strainer (Corning, Inc.; Corning, NY, USA; 352340). All cells were cultured at 37 °C with 5% CO_2_.

### 4.5. Flow Cytometry and FACS Isolation

Isolated cells from mammary glands and bone marrow were stained at 4 °C for 30 min with Fixable Viability Dye eFluor 780 (1:1000; ThermoFisher; Waltham, MA, USA; 65-0865) followed by blocking of the Fc receptors with unconjugated CD16/32 antibodies at 4 °C for 20 min (0.5 ng/µL; ThermoFisher; Waltham, MA, USA; 14-0161-85). Cells were resuspended at 1 × 10^7^/mL, then incubated with BV421 CD34 (16 ng/µL; BD Biosciences; San Jose, CA, USA; 562608), APC CD11b (2.5 ng/µL; ThermoFisher; Waltham, MA, USA; 17-0112-82), and PE CD45 (2.5 ng/µL; ThermoFisher; Waltham, MA, USA; 12-0451-82) in PBS with 2% FBS at 4 °C for 45 min. Bone marrow cells isolated from DT-treated mice were stained with Fixable Viability Dye, blocked with CD16/32 antibodies, then incubated with APC CD11b antibodies. Flow cytometry was performed using a BD LSRFortessa (BD Biosciences; San Jose, CA, USA). Fluorescence-activated cell sorting (FACS) was performed on a BD FACS Aria III cell sorter (BD Biosciences; San Jose, CA, USA). Gates were set using fluorescence-minus-one controls. Flow cytometry data from early time point transplanted mammary glands were analyzed with a fold change relative to cells isolated from SVF/EV transplants. Data were analyzed using FlowJo software package version 10 (Becton, Dickinson and Company; Ashland, OR, USA).

### 4.6. Magnetic Bead Sorting and Fibrocyte Culture

For magnetic bead sorting, CD11b antibody (0.1 µg/µL; ThermoFisher; Waltham, MA, USA; 14-0112-85) was rotated with magnetic beads (ThermoFisher; Waltham, MA, USA; 11035) at 4 °C for 30 min. Beads were washed and resuspended, then incubated with dissociated cells resuspended at a concentration of 1 × 10^7^ cells/mL in PBS supplemented with 0.1% BSA and 2 mM EDTA with 100 µL beads/1 × 10^7^ cells. Cells and beads were mixed with rotation at 4 °C for 20 min. Bead-conjugated cells were isolated with a magnet, then plated at 20,000 cells/well on 24-well plates. Cells were treated with PBS or imatinib mesylate (AdooQ Bioscience, Irvine, CA, USA; A10468) or DMSO or ISCK03 (Stem Cell Technologies, Vancouver, BC, Canada; 73732) at 5 µM, 10 µM, or 20 µM for 10 days, with a media/treatment change on day 5. Cells were fixed in 100% cold methanol for 15 min at −20 °C followed by staining with 0.1% crystal violet. Wells were imaged with a Keyence BZ-X710 microscope, and ImageJ software was used to quantify both colony number and colony size. FACS-isolated myeloid progenitor cells (CD45 + CD11b + CD34 +) and monocytes/macrophages (CD45 + CD11b + CD34-) from developing mammary tumors were plated at 500 cells/well on 8-well chamber slides (ThermoFisher; 15434) coated with poly-L-lysine (Sigma-Aldrich, St. Louis, MO, USA; P4707) and grown for 10 days. All sorted cells were grown in triplicate in MesenCult Expansion Kit Media (StemCell Technologies, Vancouver, BC, Canada; 05513) in at least 3 experiments.

### 4.7. Histology and Immunofluorescence

Tissue was paraffin-embedded, sectioned, and stained with hematoxylin and eosin (H&E) or Masson’s trichrome by the Experimental Animal Pathology Laboratory (Carbone Cancer Center, University of Wisconsin-Madison; Madison, WI, USA). Picrosirius red staining was completed as described [47]. Immunofluorescence was performed as described [103]. Primary antibodies included F4/80 (1:250; Biolegend, San Diego, CA, USA; 123101), α-smooth muscle actin (SMA; 1:5000; Sigma-Aldrich; St. Louis, MO, USA; A5228), collagen I (1:500; Abcam; Cambridge, MA, USA; ab34710), Ki67 (1:200; Abcam; ab15580), platelet-derived growth factor receptor alpha (PDGFRα; 1:200; ThermoFisher; Waltham MA, USA; PA5-16742), CD11b (1:100; Novus; Centennial, CO, USA; NB110-89474), green fluorescent protein (GFP; 1:200; Abcam; Cambridge, MA, USA; ab13970), red fluorescent protein (RFP; 1:50; Rockland Immunochemicals, Inc.; Limerick, PA, USA; 200-101-379), cytokeratin 8 (CK8; 1:500; Agilent; Santa Clara, CA, USA; M3652), cytokeratin 14 (CK14; 1:500; ThermoFisher; Waltham, MA, USA; RB-9020), SV40 T antigen (1:250; Santa Cruz Biotechnology; Dallas, TX, USA; sc-147). Secondary antibodies were obtained from ThermoFisher (Waltham, MA, USA) and included Alexa Fluor 488 goat anti-mouse IgG (A11001), Alexa Fluor 488 goat anti-rat IgG (A11006), Alexa Fluor 546 goat anti-mouse IgG (A11030), Alexa Fluor 546 goat anti-rabbit IgG (A11010). Sections were counterstained with 25 µg/mL 4′,6-diamidino-2-phenylindole (DAPI) for 10 min. Slides were then treated with the TrueView Autofluorescence Quenching Kit (Vector Laboratories; Burlingame, CA, USA; SP-8400) at room temperature for 5 min, according to the manufacturer’s instructions, and mounted using Vectashield Vibrance Antifade Mounting Medium (Vector Laboratories; Burlingame, CA, USA; H-1700).

Tissue sections were blinded and then imaged using a Leica TCS SP8 Confocal Microscope (Leica Microsystems, Buffalo Grove, IL, USA) or Nikon Eclipse E600 Microscope and QICAM Fast 1394 camera (Nikon Instruments, Inc., Melville, NY, USA). Stained slides for quantification were stained and imaged in parallel with identical image acquisition settings and exposure times. Five images were taken from each tissue section. Area of staining was quantified using Image J software using the whole image. Staining was assessed by setting a threshold using the thresholding tool. Thresholding tool settings were set and duplicated on all tissues in the groups compared. For Ki67 staining, labeled nuclei and total nuclei were counted in each image using the cell counter plug-in. For fibrocyte colonies, the freehand selection tool was used to trace the border of crystal violet-stained colonies, followed by use of the analyze/measure function.

### 4.8. Quantitative RT-PCR

Dissociated tumor cells from primary SVF/EV and SVF/CCL2 tumors were isolated based on GFP expression using FACS. RNA was isolated from tumor cells using Qiagen RNeasy Mini Kit (Qiagen; Germantown, MD, USA; 74104). RNA was reverse transcribed using the High Capacity cDNA Reverse Transcription Kit (Applied Biosciences, Waltham, MA, USA; 4368814) and Techne Thermal Cycler. Quantitative PCR was performed using iTaq SYBR Green Supermix (Bio-Rad; 172-5121) with a Bio-Rad CFX Connect Real-Time PCR Detection System (Bio-Rad; Hercules, CA, USA). Transcripts were normalized to housekeeping gene GAPDH. Data were analyzed using the ∆Cq method. Primers for CCL2 are F: 5′ GAGAGGCTGAGACTAACCCAGA and R: 5′ATCACAGCTTCTTTGGGACACT, and GAPDH are F: 5′ GAGTCAACGGATTTGGTCGT and R: 5′ TTGATTTTGGAGGGATCTCG.

### 4.9. Statistical Analysis

*p*-Values of 0.05 or less were considered significant. Data were tested for normality using Bartlett’s test prior to further statistical analysis. Error bars represent mean ± s.e.m. unless otherwise stated. Statistical analyses were conducted using GraphPad Prism v.8 (GraphPad Software, San Diego, CA, USA).

## 5. Conclusions

Obesity is significantly correlated with diminished breast cancer responses to chemotherapy, which may occur in part due to increased breast tumor desmoplasia. Under conditions of obesity, adipocytes increase CCL2 expression, leading to elevated recruitment of inflammatory macrophages. Within the tumor microenvironment, we observed that elevated CCL2 expression enhanced recruitment of macrophages during early tumor growth, followed temporally by significant increases in CAF formation and collagen deposition. Depletion of macrophages led to significant reductions in tumor growth, CAF number, and collagen deposition. Understanding how macrophages contribute to CAF formation during early tumor progression may lead to new chemoprevention strategies to reduce breast tumor growth and progression for at-risk obese women. We also identified that CCL2 expression increased recruitment of myeloid progenitor cells into growing tumors. The myeloid progenitor cell population contained fibrocytes, which were inhibited by the tyrosine kinase inhibitor, imatinib mesylate in vitro. Future studies to identify the contribution of fibrocytes to breast tumor desmoplasia may enhance therapeutic strategies to reduce tumor fibrosis and increase chemotherapeutic responses for obese breast cancer patients.

## Supplementary Materials

The following are available online at https://www.mdpi.com/2072-6694/12/8/2083/s1, Figure S1: Similar expression of oncogenes and luminal/basal epithelial cell markers in SVF/EV and SVF/CCL2 tumors from mice, Figure S2: Characterization and flow cytometry gating strategy of transplanted mammary glands, Figure S3: Gating strategy for flow cytometry of bone marrow, Figure S4: Depletion of CD11b + cells following DT treatment and end-stage tumors in Mac/SCID mice, Figure S5: CCR2 and CD11b-expressing cells in CAF populations.

## References

[1] WHO Obesity and Overweight. http://www.who.int/news-room/fact-sheets/detail/obesity-and-overweight.

[2] Calle E.E., Kaaks R. (2004). Overweight, obesity and cancer: Epidemiological evidence and proposed mechanisms. Nat. Rev. Cancer.

[3] Majed B., Moreau T., Senouci K., Salmon R.J., Fourquet A., Asselain B. (2008). Is obesity an independent prognosis factor in woman breast cancer?. Breast Cancer Res. Treat..

[4] Ewertz M., Jensen M.B., Gunnarsdottir K.A., Hojris I., Jakobsen E.H., Nielsen D., Stenbygaard L.E., Tange U.B., Cold S. (2011). Effect of obesity on prognosis after early-stage breast cancer. J. Clin. Oncol..

[5] Seo B.R., Bhardwaj P., Choi S., Gonzalez J., Andresen Eguiluz R.C., Wang K., Mohanan S., Morris P.G., Du B., Zhou X.K. (2015). Obesity-dependent changes in interstitial ECM mechanics promote breast tumorigenesis. Sci. Tansl. Med..

[6] Beck A.H., Sangoi A.R., Leung S., Marinelli R.J., Nielsen T.O., van de Vijver M.J., West R.B., van de Rijn M., Koller D. (2011). Systematic analysis of breast cancer morphology uncovers stromal features associated with survival. Sci. Tansl. Med..

[7] De Kruijf E.M., van Nes J.G., van de Velde C.J., Putter H., Smit V.T., Liefers G.J., Kuppen P.J., Tollenaar R.A., Mesker W.E. (2011). Tumor-stroma ratio in the primary tumor is a prognostic factor in early breast cancer patients, especially in triple-negative carcinoma patients. Breast Cancer Res. Treat..

[8] Dekker T.J., van de Velde C.J., van Pelt G.W., Kroep J.R., Julien J.P., Smit V.T., Tollenaar R.A., Mesker W.E. (2013). Prognostic significance of the tumor-stroma ratio: Validation study in node-negative premenopausal breast cancer patients from the EORTC perioperative chemotherapy (POP) trial (10854). Breast Cancer Res. Treat..

[9] Moorman A.M., Vink R., Heijmans H.J., van der Palen J., Kouwenhoven E.A. (2012). The prognostic value of tumour-stroma ratio in triple-negative breast cancer. Eur. J. Surg. Oncol..

[10] Weisberg S.P., McCann D., Desai M., Rosenbaum M., Leibel R.L., Ferrante A.W. (2003). Obesity is associated with macrophage accumulation in adipose tissue. J. Clin. Investig..

[11] Murano I., Barbatelli G., Parisani V., Latini C., Muzzonigro G., Castellucci M., Cinti S. (2008). Dead adipocytes, detected as crown-like structures, are prevalent in visceral fat depots of genetically obese mice. J. Lipid Res..

[12] Subbaramaiah K., Howe L.R., Bhardwaj P., Du B., Gravaghi C., Yantiss R.K., Zhou X.K., Blaho V.A., Hla T., Yang P. (2011). Obesity is associated with inflammation and elevated aromatase expression in the mouse mammary gland. Cancer Prev. Res. (Phila.).

[13] Sartipy P., Loskutoff D.J. (2003). Monocyte chemoattractant protein 1 in obesity and insulin resistance. Proc. Natl. Acad. Sci. USA.

[14] Chen A., Mumick S., Zhang C., Lamb J., Dai H., Weingarth D., Mudgett J., Chen H., MacNeil D.J., Reitman M.L. (2005). Diet induction of monocyte chemoattractant protein-1 and its impact on obesity. Obes. Res..

[15] Kanda H., Tateya S., Tamori Y., Kotani K., Hiasa K., Kitazawa R., Kitazawa S., Miyachi H., Maeda S., Egashira K. (2006). MCP-1 contributes to macrophage infiltration into adipose tissue, insulin resistance, and hepatic steatosis in obesity. J. Clin. Investig..

[16] Weisberg S.P., Hunter D., Huber R., Lemieux J., Slaymaker S., Vaddi K., Charo I., Leibel R.L., Ferrante A.W. (2006). CCR2 modulates inflammatory and metabolic effects of high-fat feeding. J. Clin. Investig..

[17] Ueno T., Toi M., Saji H., Muta M., Bando H., Kuroi K., Koike M., Inadera H., Matsushima K. (2000). Significance of macrophage chemoattractant protein-1 in macrophage recruitment, angiogenesis, and survival in human breast cancer. Clin. Cancer Res..

[18] Arendt L.M., McCready J., Keller P.J., Baker D.D., Naber S.P., Seewaldt V., Kuperwasser C. (2013). Obesity promotes breast cancer by CCL2-mediated macrophage recruitment and angiogenesis. Cancer Res..

[19] Fujimoto H., Sangai T., Ishii G., Ikehara A., Nagashima T., Miyazaki M., Ochiai A. (2009). Stromal MCP-1 in mammary tumors induces tumor-associated macrophage infiltration and contributes to tumor progression. J. Int. Cancer.

[20] Matsui Y., Tomaru U., Miyoshi A., Ito T., Fukaya S., Miyoshi H., Atsumi T., Ishizu A. (2014). Overexpression of TNF-alpha converting enzyme promotes adipose tissue inflammation and fibrosis induced by high fat diet. Exp. Mol. Pathol..

[21] Tanaka M., Ikeda K., Suganami T., Komiya C., Ochi K., Shirakawa I., Hamaguchi M., Nishimura S., Manabe I., Matsuda T. (2014). Macrophage-inducible C-type lectin underlies obesity-induced adipose tissue fibrosis. Nat. Commun..

[22] Buechler C., Krautbauer S., Eisinger K. (2015). Adipose tissue fibrosis. World J. Diabetes.

[23] Guglielmi V., Cardellini M., Cinti F., Corgosinho F., Cardolini I., D’Adamo M., Zingaretti M.C., Bellia A., Lauro D., Gentileschi P. (2015). Omental adipose tissue fibrosis and insulin resistance in severe obesity. Nutr. Diabetes.

[24] Sun X., Glynn D.J., Hodson L.J., Huo C., Britt K., Thompson E.W., Woolford L., Evdokiou A., Pollard J.W., Robertson S.A. (2017). CCL2-driven inflammation increases mammary gland stromal density and cancer susceptibility in a transgenic mouse model. Breast Cancer Res..

[25] Tsuyada A., Chow A., Wu J., Somlo G., Chu P., Loera S., Luu T., Li A.X., Wu X., Ye W. (2012). CCL2 mediates cross-talk between cancer cells and stromal fibroblasts that regulates breast cancer stem cells. Cancer Res..

[26] Yao M., Fang W., Smart C., Hu Q., Huang S., Alvarez N., Fields P., Cheng N. (2019). CCR2 Chemokine Receptors Enhance Growth and Cell-Cycle Progression of Breast Cancer Cells through SRC and PKC Activation. Mol. Cancer Res..

[27] Sugimoto H., Mundel T.M., Kieran M.W., Kalluri R. (2006). Identification of fibroblast heterogeneity in the tumor microenvironment. Cancer Biol. Ther..

[28] Augsten M. (2014). Cancer-associated fibroblasts as another polarized cell type of the tumor microenvironment. Front. Oncol..

[29] Cortez E., Roswall P., Pietras K. (2014). Functional subsets of mesenchymal cell types in the tumor microenvironment. Semin. Cancer Biol..

[30] Paulsson J., Micke P. (2014). Prognostic relevance of cancer-associated fibroblasts in human cancer. Semin. Cancer Biol..

[31] Houthuijzen J.M., Jonkers J. (2018). Cancer-associated fibroblasts as key regulators of the breast cancer tumor microenvironment. Cancer Metastasis Rev..

[32] Outzen H.C., Custer R.P. (1975). Growth of human normal and neoplastic mammary tissues in the cleared mammary fat pad of the nude mouse. J. Natl. Cancer Inst..

[33] McManus M.J., Welsch C.W. (1980). DNA synthesis of benign human breast tumors in the untreated athymic “nude” mouse. An in vivo model to study hormonal influences on growth of human breast tissues. Cancer.

[34] Arendt L.M., Keller P.J., Skibinski A., Goncalves K., Naber S.P., Buchsbaum R.J., Gilmore H., Come S.E., Kuperwasser C. (2014). Anatomical localization of progenitor cells in human breast tissue reveals enrichment of uncommitted cells within immature lobules. Breast Cancer Res..

[35] Proia T.A., Keller P.J., Gupta P.B., Klebba I., Jones A.D., Sedic M., Gilmore H., Tung N., Naber S.P., Schnitt S. (2011). Genetic predisposition directs breast cancer phenotype by dictating progenitor cell fate. Cell Stem. Cell.

[36] Keller P.J., Arendt L.M., Skibinski A., Logvinenko T., Klebba I., Dong S., Smith A.E., Prat A., Perou C.M., Gilmore H. (2012). Defining the cellular precursors to human breast cancer. Proc. Natl. Acad. Sci. USA.

[37] Hillers L.E., D’Amato J.V., Chamberlin T., Paderta G., Arendt L.M. (2018). Obesity-Activated Adipose-Derived Stromal Cells Promote Breast Cancer Growth and Invasion. Neoplasia.

[38] Kuperwasser C., Chavarria T., Wu M., Magrane G., Gray J.W., Carey L., Richardson A., Weinberg R.A. (2004). Reconstruction of functionally normal and malignant human breast tissues in mice. Proc. Natl. Acad. Sci. USA.

[39] Keeley E.C., Mehrad B., Strieter R.M. (2010). Fibrocytes: Bringing new insights into mechanisms of inflammation and fibrosis. Int. J. Biochem. Cell Biol..

[40] Reilkoff R.A., Bucala R., Herzog E.L. (2011). Fibrocytes: Emerging effector cells in chronic inflammation. Nat. Rev. Immunol..

[41] Cao T., Rajasingh S., Rajasingh J. (2016). Circulating fibrocytes serve as a marker for clinical diagnosis. Ann. Transl. Med..

[42] Arendt L.M., Rudnick J.A., Keller P.J., Kuperwasser C. (2010). Stroma in breast development and disease. Semin. Cell Dev. Biol..

[43] Hu M., Yao J., Carroll D.K., Weremowicz S., Chen H., Carrasco D., Richardson A., Violette S., Nikolskaya T., Nikolsky Y. (2008). Regulation of in situ to invasive breast carcinoma transition. Cancer Cell.

[44] Osuala K.O., Sameni M., Shah S., Aggarwal N., Simonait M.L., Franco O.E., Hong Y., Hayward S.W., Behbod F., Mattingly R.R. (2015). Il-6 signaling between ductal carcinoma in situ cells and carcinoma-associated fibroblasts mediates tumor cell growth and migration. BMC Cancer.

[45] Cailhier J.F., Partolina M., Vuthoori S., Wu S., Ko K., Watson S., Savill J., Hughes J., Lang R.A. (2005). Conditional macrophage ablation demonstrates that resident macrophages initiate acute peritoneal inflammation. J. Immunol..

[46] Stoneman V., Braganza D., Figg N., Mercer J., Lang R., Goddard M., Bennett M. (2007). Monocyte/macrophage suppression in CD11b diphtheria toxin receptor transgenic mice differentially affects atherogenesis and established plaques. Circ. Res..

[47] Wegner K.A., Keikhosravi A., Eliceiri K.W., Vezina C.M. (2017). Fluorescence of Picrosirius Red Multiplexed With Immunohistochemistry for the Quantitative Assessment of Collagen in Tissue Sections. J. Histochem. Cytochem. Off. J. Histochem. Soc..

[48] Fujimura N., Xu B., Dalman J., Deng H., Aoyama K., Dalman R.L. (2015). CCR2 inhibition sequesters multiple subsets of leukocytes in the bone marrow. Sci. Rep..

[49] Aono Y., Kishi M., Yokota Y., Azuma M., Kinoshita K., Takezaki A., Sato S., Kawano H., Kishi J., Goto H. (2014). Role of platelet-derived growth factor/platelet-derived growth factor receptor axis in the trafficking of circulating fibrocytes in pulmonary fibrosis. Am. J. Respir. Cell Mol. Biol..

[50] Distler J.H., Distler O. (2009). Imatinib as a novel therapeutic approach for fibrotic disorders. Rheumatology.

[51] Osumi T., Miharu M., Tanaka R., Du W., Takahashi T., Shimada H. (2012). Imatinib is effective for prevention and improvement of fibrotic fasciitis as a manifestation of chronic GVHD. Bone Marrow Trans..

[52] Vittal R., Zhang H., Han M.K., Moore B.B., Horowitz J.C., Thannickal V.J. (2007). Effects of the protein kinase inhibitor, imatinib mesylate, on epithelial/mesenchymal phenotypes: Implications for treatment of fibrotic diseases. J. Pharmacol. Exp. Ther..

[53] Bucala R., Spiegel L.A., Chesney J., Hogan M., Cerami A. (1994). Circulating fibrocytes define a new leukocyte subpopulation that mediates tissue repair. Mol. Med..

[54] Bellows C.F., Zhang Y., Simmons P.J., Khalsa A.S., Kolonin M.G. (2011). Influence of BMI on level of circulating progenitor cells. Obesity (Silver Spring, MD).

[55] Terai S., Fushida S., Tsukada T., Kinoshita J., Oyama K., Okamoto K., Makino I., Tajima H., Ninomiya I., Fujimura T. (2015). Bone marrow derived “fibrocytes” contribute to tumor proliferation and fibrosis in gastric cancer. Gastric Cancer.

[56] Mitsuhashi A., Goto H., Saijo A., Trung V.T., Aono Y., Ogino H., Kuramoto T., Tabata S., Uehara H., Izumi K. (2015). Fibrocyte-like cells mediate acquired resistance to anti-angiogenic therapy with bevacizumab. Nat. Commun..

[57] Eiro N., Fernandez-Garcia B., Vazquez J., Del Casar J.M., Gonzalez L.O., Vizoso F.J. (2015). A phenotype from tumor stroma based on the expression of metalloproteases and their inhibitors, associated with prognosis in breast cancer. Oncoimmunology.

[58] Park S.Y., Kim H.M., Koo J.S. (2015). Differential expression of cancer-associated fibroblast-related proteins according to molecular subtype and stromal histology in breast cancer. Breast Cancer Res. Treat..

[59] Niedermeier M., Reich B., Rodriguez Gomez M., Denzel A., Schmidbauer K., Gobel N., Talke Y., Schweda F., Mack M. (2009). CD4+ T cells control the differentiation of Gr1+ monocytes into fibrocytes. Proc. Natl. Acad. Sci. USA.

[60] Eto H., Ishimine H., Kinoshita K., Watanabe-Susaki K., Kato H., Doi K., Kuno S., Kurisaki A., Yoshimura K. (2013). Characterization of human adipose tissue-resident hematopoietic cell populations reveals a novel macrophage subpopulation with CD34 expression and mesenchymal multipotency. Stem. Cells Dev..

[61] Pilling D., Fan T., Huang D., Kaul B., Gomer R.H. (2009). Identification of markers that distinguish monocyte-derived fibrocytes from monocytes, macrophages, and fibroblasts. PLoS ONE.

[62] Suga H., Rennert R.C., Rodrigues M., Sorkin M., Glotzbach J.P., Januszyk M., Fujiwara T., Longaker M.T., Gurtner G.C. (2014). Tracking the elusive fibrocyte: Identification and characterization of collagen-producing hematopoietic lineage cells during murine wound healing. Stem. Cells.

[63] Chauhan H., Abraham A., Phillips J.R., Pringle J.H., Walker R.A., Jones J.L. (2003). There is more than one kind of myofibroblast: Analysis of CD34 expression in benign, in situ, and invasive breast lesions. J. Clin. Pathol..

[64] Barth P.J., Ebrahimsade S., Ramaswamy A., Moll R. (2002). CD34+ fibrocytes in invasive ductal carcinoma, ductal carcinoma in situ, and benign breast lesions. Virchows Arch..

[65] Joshi P.A., Waterhouse P.D., Kasaian K., Fang H., Gulyaeva O., Sul H.S., Boutros P.C., Khokha R. (2019). PDGFRα(+) stromal adipocyte progenitors transition into epithelial cells during lobulo-alveologenesis in the murine mammary gland. Nat. Commun..

[66] Dvorak H.F. (1986). Tumors: Wounds that do not heal. Similarities between tumor stroma generation and wound healing. N. Engl. J. Med..

[67] Wynn T.A. (2008). Cellular and molecular mechanisms of fibrosis. J. Pathol..

[68] Wynn T.A., Vannella K.M. (2016). Macrophages in Tissue Repair, Regeneration, and Fibrosis. Immunity.

[69] Nowicki A., Szenajch J., Ostrowska G., Wojtowicz A., Wojtowicz K., Kruszewski A.A., Maruszynski M., Aukerman S.L., Wiktor-Jedrzejczak W. (1996). Impaired tumor growth in colony-stimulating factor 1 (CSF-1)-deficient, macrophage-deficient op/op mouse: Evidence for a role of CSF-1-dependent macrophages in formation of tumor stroma. Int. J. Cancer.

[70] Afik R., Zigmond E., Vugman M., Klepfish M., Shimshoni E., Pasmanik-Chor M., Shenoy A., Bassat E., Halpern Z., Geiger T. (2016). Tumor macrophages are pivotal constructors of tumor collagenous matrix. J. Exp. Med..

[71] Madsen D.H., Jurgensen H.J., Siersbaek M.S., Kuczek D.E., Grey Cloud L., Liu S., Behrendt N., Grontved L., Weigert R., Bugge T.H. (2017). Tumor-Associated Macrophages Derived from Circulating Inflammatory Monocytes Degrade Collagen through Cellular Uptake. Cell Rep..

[72] Ingman W.V., Wyckoff J., Gouon-Evans V., Condeelis J., Pollard J.W. (2006). Macrophages promote collagen fibrillogenesis around terminal end buds of the developing mammary gland. Dev. Dyn..

[73] O’Brien J., Lyons T., Monks J., Lucia M.S., Wilson R.S., Hines L., Man Y.G., Borges V., Schedin P. (2010). Alternatively activated macrophages and collagen remodeling characterize the postpartum involuting mammary gland across species. Am. J. Pathol..

[74] Abe R., Donnelly S.C., Peng T., Bucala R., Metz C.N. (2001). Peripheral Blood Fibrocytes: Differentiation Pathway and Migration to Wound Sites. J. Immun..

[75] Assoian R.K., Fleurdelys B.E., Stevenson H.C., Miller P.J., Madtes D.K., Raines E.W., Ross R., Sporn M.B. (1987). Expression and secretion of type beta transforming growth factor by activated human macrophages. Proc. Natl. Acad. Sci. USA.

[76] Pilling D., Vakil V., Cox N., Gomer R.H. (2015). TNF-alpha-stimulated fibroblasts secrete lumican to promote fibrocyte differentiation. Proc. Natl. Acad. Sci. USA.

[77] Meng X.M., Wang S., Huang X.R., Yang C., Xiao J., Zhang Y., To K.F., Nikolic-Paterson D.J., Lan H.Y. (2016). Inflammatory macrophages can transdifferentiate into myofibroblasts during renal fibrosis. Cell Death Dis..

[78] Wang Y.Y., Jiang H., Pan J., Huang X.R., Wang Y.C., Huang H.F., To K.F., Nikolic-Paterson D.J., Lan H.Y., Chen J.H. (2017). Macrophage-to-Myofibroblast Transition Contributes to Interstitial Fibrosis in Chronic Renal Allograft Injury. J. Am. Soc. Nephrol..

[79] Wang S., Meng X.M., Ng Y.Y., Ma F.Y., Zhou S., Zhang Y., Yang C., Huang X.R., Xiao J., Wang Y.Y. (2015). TGF-β/Smad3 signalling regulates the transition of bone marrow-derived macrophages into myofibroblasts during tissue fibrosis. Oncotarget.

[80] Lin E.Y., Li J.F., Gnatovskiy L., Deng Y., Zhu L., Grzesik D.A., Qian H., Xue X.N., Pollard J.W. (2006). Macrophages regulate the angiogenic switch in a mouse model of breast cancer. Cancer Res..

[81] Lu H., Clauser K.R., Tam W.L., Frose J., Ye X., Eaton E.N., Reinhardt F., Donnenberg V.S., Bhargava R., Carr S.A. (2014). A breast cancer stem cell niche supported by juxtacrine signalling from monocytes and macrophages. Nat. Cell Biol..

[82] Mitchem J.B., Brennan D.J., Knolhoff B.L., Belt B.A., Zhu Y., Sanford D.E., Belaygorod L., Carpenter D., Collins L., Piwnica-Worms D. (2013). Targeting tumor-infiltrating macrophages decreases tumor-initiating cells, relieves immunosuppression, and improves chemotherapeutic responses. Cancer Res..

[83] Lindsten T., Hedbrant A., Ramberg A., Wijkander J., Solterbeck A., Eriksson M., Delbro D., Erlandsson A. (2017). Effect of macrophages on breast cancer cell proliferation, and on expression of hormone receptors, uPAR and HER-2. Int. J. Oncol..

[84] Gao M.Q., Kim B.G., Kang S., Choi Y.P., Yoon J.H., Cho N.H. (2013). Human breast cancer-associated fibroblasts enhance cancer cell proliferation through increased TGF-α cleavage by ADAM17. Cancer Lett..

[85] Suh J., Kim D.H., Lee Y.H., Jang J.H., Surh Y.J. (2020). Fibroblast growth factor-2, derived from cancer-associated fibroblasts, stimulates growth and progression of human breast cancer cells via FGFR1 signaling. Mol. Carcinog..

[86] Stowers R.S., Allen S.C., Sanchez K., Davis C.L., Ebelt N.D., Van Den Berg C., Suggs L.J. (2017). Extracellular Matrix Stiffening Induces a Malignant Phenotypic Transition in Breast Epithelial Cells. Cell Mol. Bioeng..

[87] Taylor M.A., Amin J.D., Kirschmann D.A., Schiemann W.P. (2011). Lysyl oxidase contributes to mechanotransduction-mediated regulation of transforming growth factor-β signaling in breast cancer cells. Neoplasia.

[88] Provenzano P.P., Keely P.J. (2011). Mechanical signaling through the cytoskeleton regulates cell proliferation by coordinated focal adhesion and Rho GTPase signaling. J. Cell Sci..

[89] Maass N., Schem C., Bauerschlag D.O., Tiemann K., Schaefer F.W., Hanson S., Muth M., Baier M., Weigel M.T., Wenners A.S. (2014). Final safety and efficacy analysis of a phase I/II trial with imatinib and vinorelbine for patients with metastatic breast cancer. Oncology.

[90] Pishvaian M.J., Slack R., Koh E.Y., Beumer J.H., Hartley M.L., Cotarla I., Deeken J., He A.R., Hwang J., Malik S. (2012). A Phase I clinical trial of the combination of imatinib and paclitaxel in patients with advanced or metastatic solid tumors refractory to standard therapy. Cancer Chemother. Pharmacol..

[91] Yardley D.A., Burris H.A., Markus T., Spigel D.R., Greco F.A., Mainwaring M., Waterhouse D.M., Webb C.D., Hainsworth J.D. (2009). Phase II trial of docetaxal plus imatinib mesylate in the treatment of patients with metastatic breast cancer. Clin. Breast Cancer.

[92] Lipton A., Campbell-Baird C., Harvey H., Kim C., Demers L., Costa L. (2010). Phase I trial of zoledronic acid + imatinib mesylate (Gleevec) in patients with bone metastases. Am. J. Clin. Oncol..

[93] Chew H.K., Barlow W.E., Albain K., Lew D., Gown A., Hayes D.F., Gralow J., Hortobagyi G.N., Livingston R. (2008). A phase II study of imatinib mesylate and capecitabine in metastatic breast cancer: Southwest Oncology Group Study 0338. Clin. Breast Cancer.

[94] Cristofanilli M., Morandi P., Krishnamurthy S., Reuben J.M., Lee B.N., Francis D., Booser D.J., Green M.C., Arun B.K., Pusztai L. (2008). Imatinib mesylate (Gleevec) in advanced breast cancer-expressing C-Kit or PDGFR-beta: Clinical activity and biological correlations. Ann. Oncol..

[95] Modi S., Seidman A.D., Dickler M., Moasser M., D’Andrea G., Moynahan M.E., Menell J., Panageas K.S., Tan L.K., Norton L. (2005). A phase II trial of imatinib mesylate monotherapy in patients with metastatic breast cancer. Breast Cancer Res. Treat..

[96] Gordon J., Spiera R. (2011). Imatinib and the treatment of fibrosis: Recent trials and tribulations. Curr. Rheumatol. Rep..

[97] Gioni V., Karampinas T., Voutsinas G., Roussidis A.E., Papadopoulos S., Karamanos N.K., Kletsas D. (2008). Imatinib mesylate inhibits proliferation and exerts an antifibrotic effect in human breast stroma fibroblasts. Mol. Cancer Res..

[98] Nishioka Y., Azuma M., Kishi M., Aono Y. (2013). Targeting platelet-derived growth factor as a therapeutic approach in pulmonary fibrosis. J. Med. Investig..

[99] Canestaro W.J., Forrester S.H., Raghu G., Ho L., Devine B.E. (2016). Drug Treatment of Idiopathic Pulmonary Fibrosis: Systematic Review and Network Meta-Analysis. Chest.

[100] Choi S.S., Kim E.S., Jung J.E., Marciano D.P., Jo A., Koo J.Y., Choi S.Y., Yang Y.R., Jang H.J., Kim E.K. (2016). PPARgamma Antagonist Gleevec Improves Insulin Sensitivity and Promotes the Browning of White Adipose Tissue. Diabetes.

[101] Fitter S., Vandyke K., Gronthos S., Zannettino A.C. (2012). Suppression of PDGF-induced PI3 kinase activity by imatinib promotes adipogenesis and adiponectin secretion. J. Mol. Endocrinol..

[102] Proia D.A., Kuperwasser C. (2006). Reconstruction of human mammary tissues in a mouse model. Nat. Protoc..

[103] Chamberlin T., D’Amato J.V., Arendt L.M. (2017). Obesity reversibly depletes the basal cell population and enhances mammary epithelial cell estrogen receptor alpha expression and progenitor activity. Breast Cancer Res..

