# Supplementary material for: Stromal CCL2 Signaling Promotes Mammary Tumor Fibrosis through Recruitment of Myeloid-Lineage Cells
Source: Cancers (Basel). 2020 Jul 28;12(8):2083. doi: 10.3390/cancers12082083 (PMC7465971; doi:10.3390/cancers12082083)
Supplement: Supplementary file 1 [file cancers-12-02083-s001.pdf]

Supplementary Materials:

Stromal CCL2 Signaling Promotes Mammary Tumor Fibrosis Through Recruitment of Myeloid-Lineage Cells

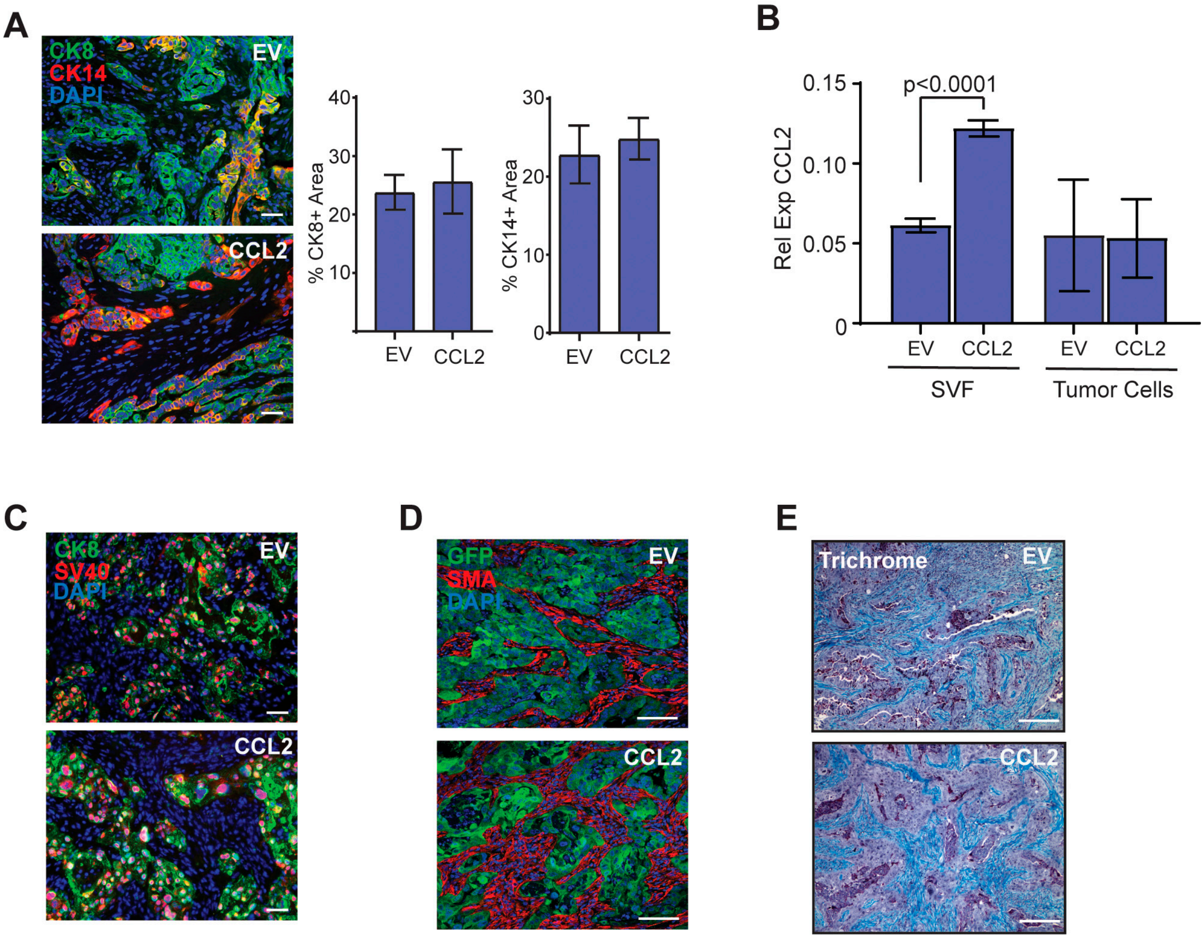

Figure S1. Similar expression of oncogenes and luminal/basal epithelial cell markers in SVF/EV and SVF/CCL2 tumors from mice. (**A**) Average area of cytokeratin 8 (CK8) and cytokeratin 14 (CK14) in end-stage tumors (n = 12 tumors/group; Student's t-test). (**B**) Relative expression of CCL2 in SVF/EV, SVF/CCL2, and tumor cells isolated from SVF/EV and SVF/CCL2 tumors. Expression normalized to GAPDH expression (n = 3 tumors/group; one-way ANOVA with Tukey's multiple comparison test). (**C**) Representative images of SV40 expression co-localized with CK8 expression in tumor cells. (**D**) Representative images of GFP+ human tumor cells and GFP- mouse stromal cells expressing SMA. (**E**) Representative images of Masson's trichrome stain of SVF/EV and SVF/CCL2 tumor sections. Bars represent mean ± s.e.m. Magnification bars = 50 μm.

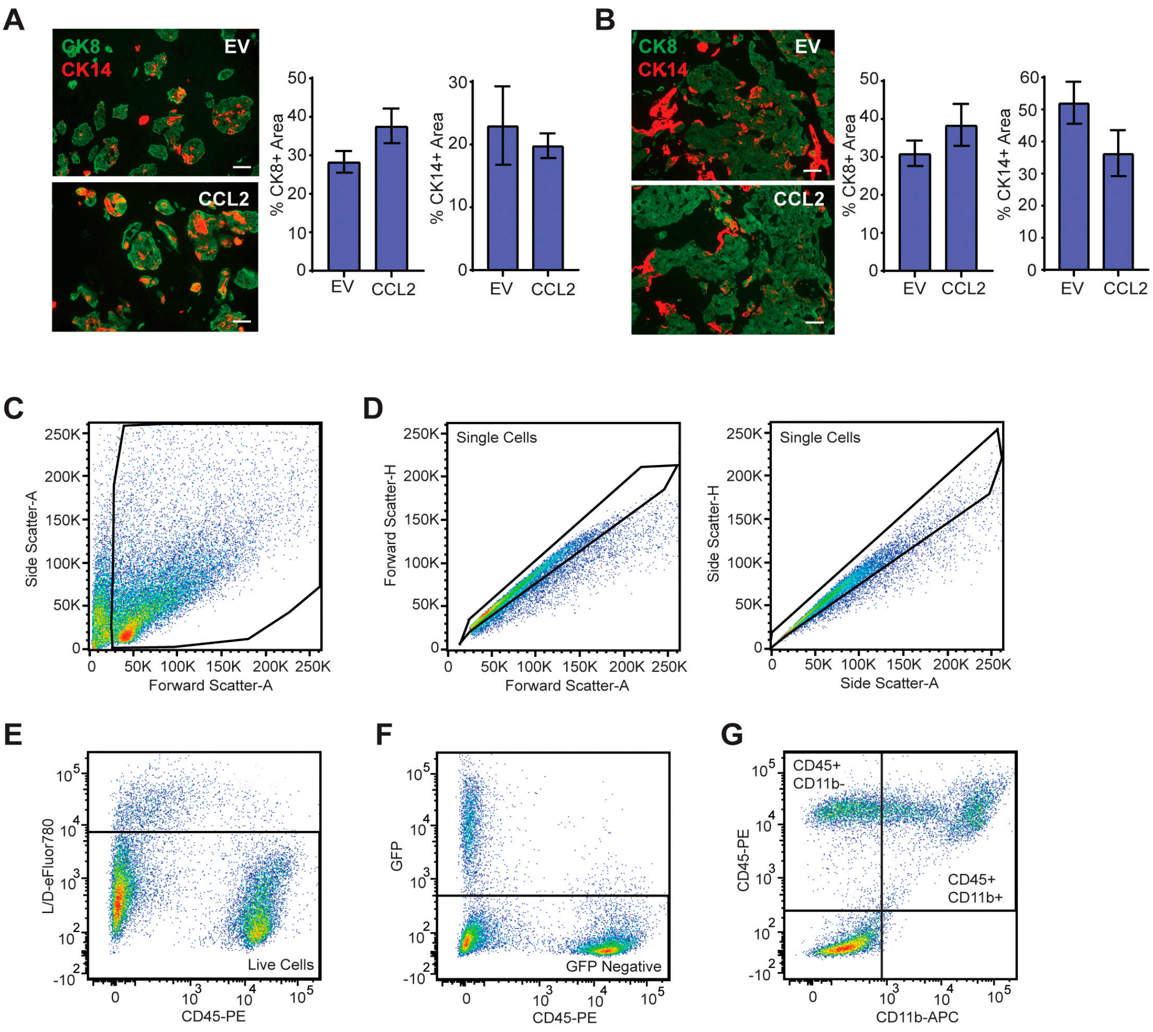

Figure S2. Characterization and flow cytometry gating strategy of transplanted mammary glands. Average area of cytokeratin 8 (CK8) and cytokeratin 14 (CK14) in 1.5 week (**A**) and 2.5 week (**B**) transplants (n = 4 transplants/group; Student's t-test). Gating strategy. (**C**) Gating out debris. (**D**) Single cells. (**E**) Live cells. (**F**) Gating out GFP+ human epithelial cells. (**G**) CD45+CD11b+ cells. Magnification bars = 50 µm.

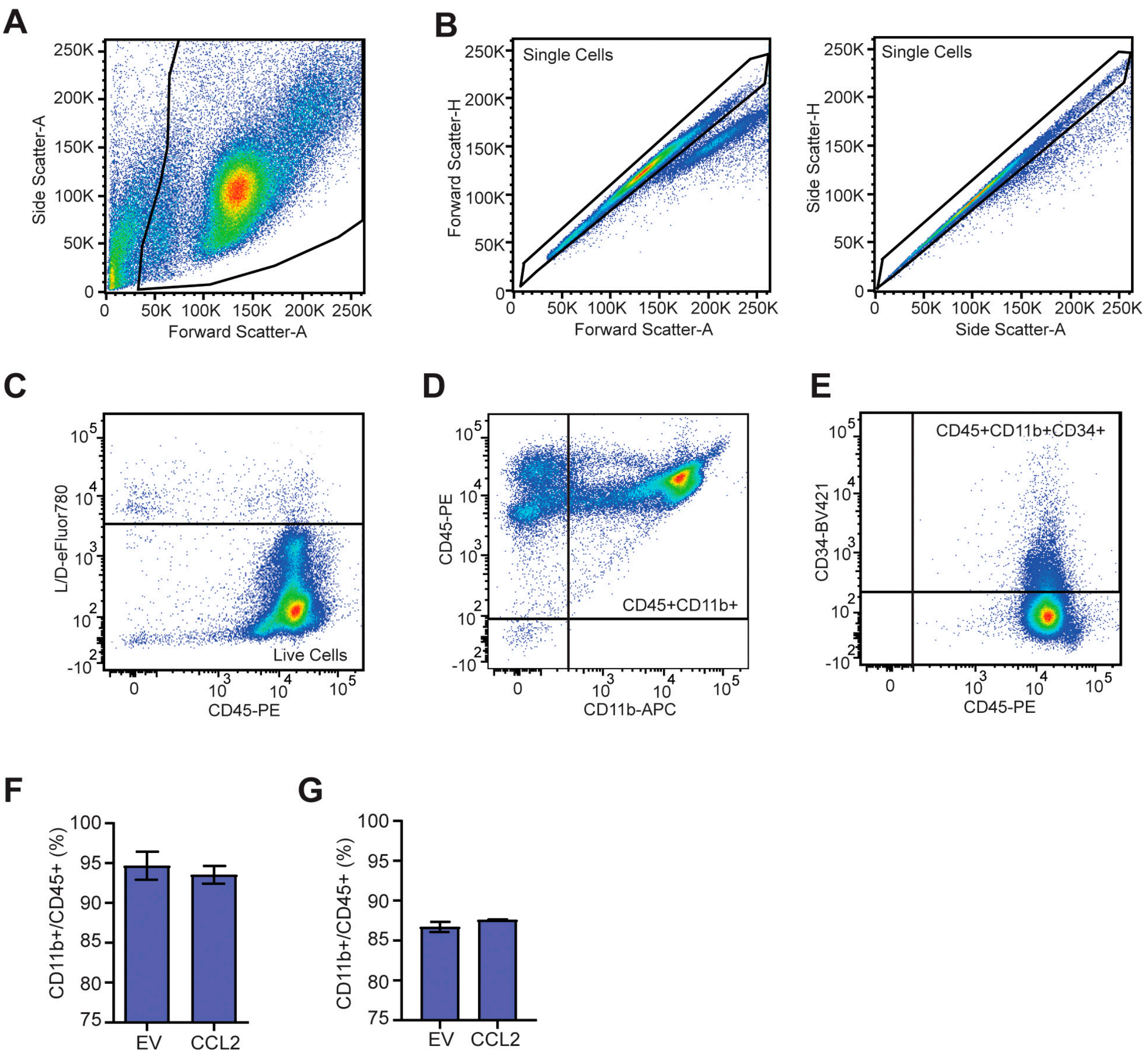

Figure S3. Gating strategy for flow cytometry of bone marrow. (**A**) Gating out debris. (**B**) Single cells. (**C**) Live cells. (**D**) CD45+CD11b+ cells. (**E**) CD45+CD11b+CD34+ cells. (**F**) CD45+CD11b+ monocytes/macrophages in the bone marrow at 1.5 weeks post-transplantation were quantified by flow cytometry (n = 6 EV, 7 CCL2; Mann-Whitney U test). (**G**) CD45+CD11b+ monocytes/macrophages in the bone marrow at 2.5 weeks post-transplantation were quantified by flow cytometry (n = 3 mice/group; Mann-Whitney U test).

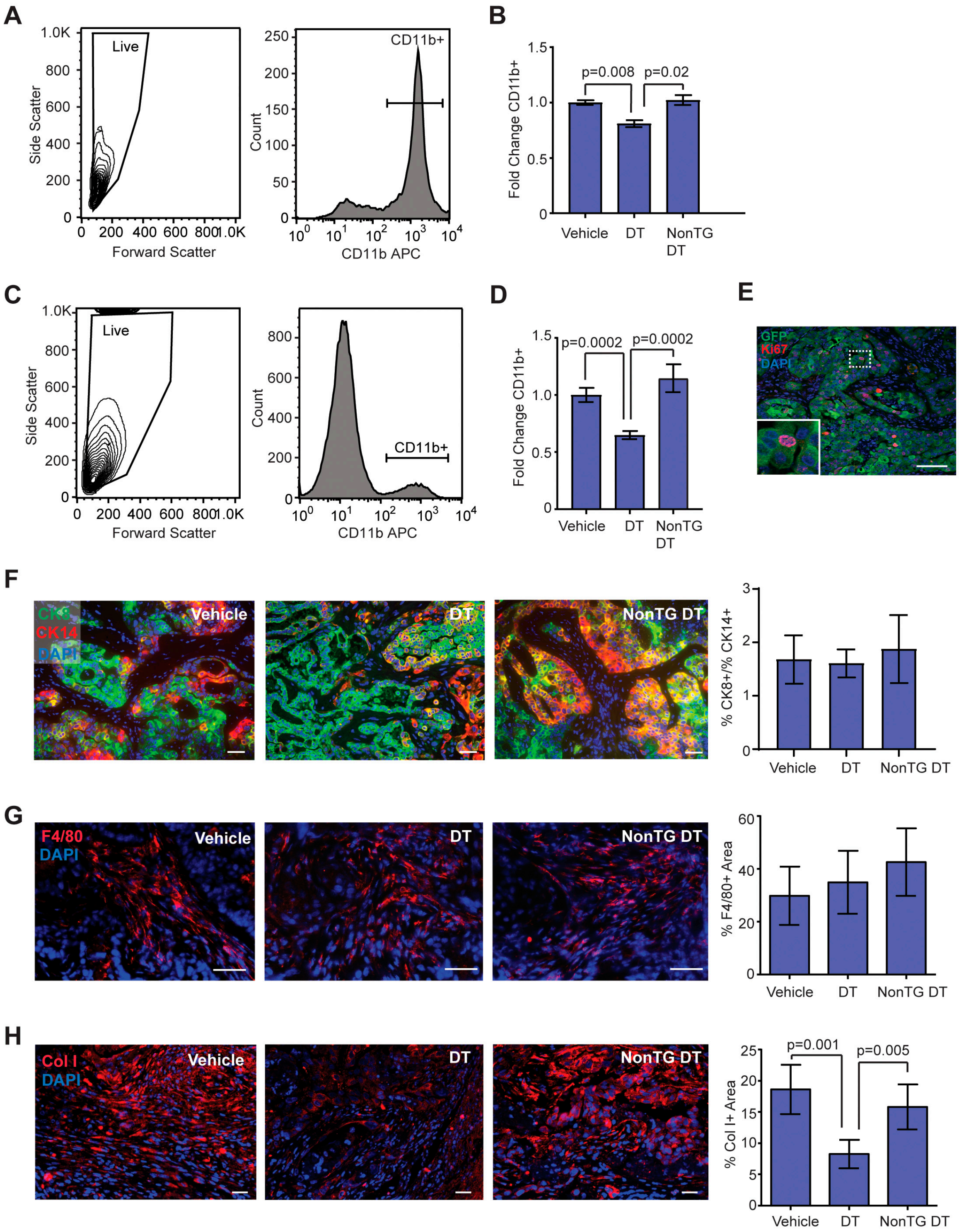

Figure S4. Depletion of CD11b+ cells following DT treatment and end-stage tumors in Mac/SCID mice. (**A**) Bone marrow cells isolated from Mac/SCID transgenic mice and nontransgenic littermates (NonTG) treated with vehicle or DT were gated for live cells followed by CD11b+ cells. (**B**) Fold change of CD11b+ cells in the bone marrow of vehicle or DT-treated mice (n = 5 mice/group; Kruskal-Wallis test with Dunn's multiple comparisons test). (**C**) Cells isolated from mammary glands of Mac/SCID transgenic mice and nontransgenic

littermates (NonTG) treated with vehicle or DT were gated for live cells followed by CD11b+ cells. (**D**) Fold change of CD11b+ cells in the mammary glands of DT or vehicle-treated mice (*n* = 5 mice/group; Kruskal-Wallis test with Dunn's multiple comparisons test). (**E**) Representative image of GFP+ human epithelial cells co-stained with Ki67. (**F**) Representative images of CK8 and CK14 staining in end-stage tumors (*n* = 8 tumors/group; one-way ANOVA with Tukey's multiple comparisons test). (**G**) Percent area of F4/80+ macrophages in end-stage tumors (*n* = 8 tumors/group; one-way ANOVA with Tukey's multiple comparisons test). (**H**) Percent area of collagen I+ cells in end-stage tumors (*n* = 8 tumors/group; one-way ANOVA with Tukey's multiple comparisons test). Magnification bars = 50 μm.

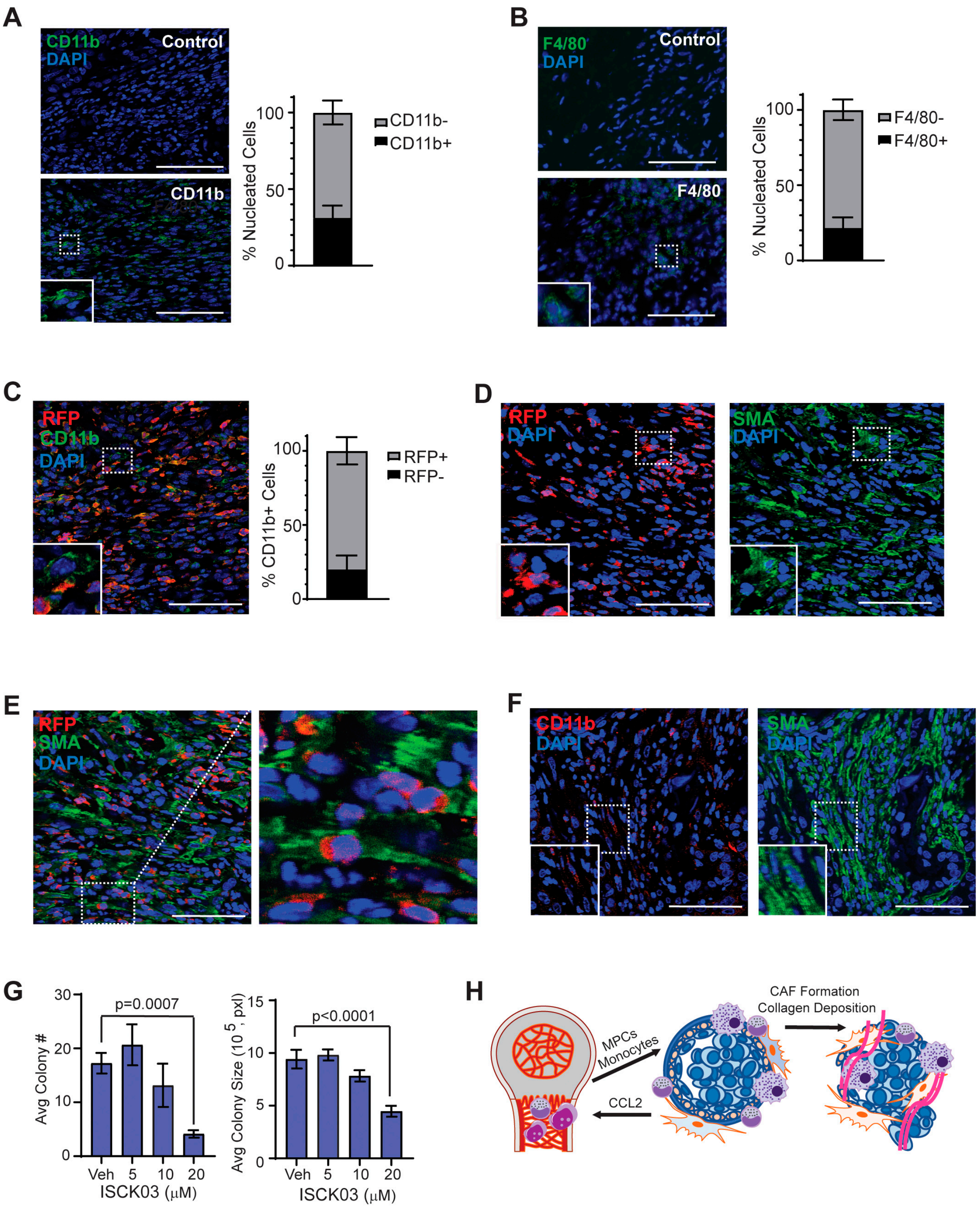

Figure S5. CCR2 and CD11b-expressing cells in CAF populations. (**A**) Percent of CD11b+ cells in total cells (n = 3 CCR2/RFP tumors). (**B**) Percent of F4/80+ cells in total cells (n = 3 CCR2/RFP tumors). (**C**) Percent of RFP+ cells in total CD11b+ expressing cells (n = 3 CCR2/RFP tumors). (**D**) Representative image of RFP+ and SMA+ cells within tumor stroma. (**E**) Magnified image of RFP+SMA+ cells within CCR2/RFP tumors. (**F**) Representative image of CD11b+ and SMA+ cells within tumor stroma. (**G**) Fibrocyte colony number and

size with vehicle, 5 μM, 10 μM, and 20 μM ISCK03 treatment (n = 2 experiments in triplicate; Kruskal-Wallis test with Dunn's multiple comparisons test). (**H**) Model of immune cell recruitment and CAF formation during transition to invasive ductal carcinoma. Magnification bars = 50 μm.
